# Mapping spatial colleague connectivity patterns from individual-level registry data to inform regional pandemic interventions

**DOI:** 10.1371/journal.pcbi.1014721

**Published:** 2026-08-31

**Authors:** PingPing Song, Sake J. de Vlas, Tom Emery, Luc E. Coffeng

**Affiliations:** 1 Department of Public Health, Erasmus MC, University Medical Center Rotterdam, Rotterdam, The Netherlands; 2 Pandemic and Disaster Preparedness Center (PDPC), Delft, Rotterdam, The Netherlands; 3 Department of Public Administration and Sociology, Erasmus University Rotterdam, Rotterdam, The Netherlands; University of Melbourne, AUSTRALIA

## Abstract

A concern in infectious disease modelling is how accurately population mixing is incorporated, as it shapes the type and frequency of contacts through which infection spreads, and consequently, estimated intervention effectiveness. Although synthesizing mixing patterns from diary-based surveys is an established framework, geographical information is poorly or sparsely captured. Here we propose a generalizable workflow to quantify geographical connectivity from job registry data covering over 8 million Dutch working population. The derived colleague connectedness shows heterogeneous spatial patterns, quantified from the number of connections per municipality triplet, two residential municipalities and one shared workplace municipality. We illustrate the epidemiological relevance of this spatial connectivity by using SARS-CoV-2 Omicron as an example: a two-fold increase in within-province connections was associated with a 3.7-day earlier (95% CI: 0.6 to 6.6 days) Omicron onset, and between-province connectivity was associated with a 2.5 days earlier (95% CI: -1.0 to 6.2 days) onset. Based on our estimates of spatial connectivity, we quantified the number of colleague connections that would be removed in case of regional mobility restrictions such as a lockdown: locking down the whole province Zeeland would remove 2.6% of colleague links at the national level while the city Amsterdam alone would remove 10.0%. In future modelling studies, these highly fine-grained spatial connectivity data could be used as spatial mixing matrices to more explicitly capture the connectedness and dependency between regions to inform more tailored policy measures.

## Author summary

Respiratory infectious disease outbreaks and pandemics pose a significant societal risk because of their direct health burden as well as the social and economic disruptions caused by measures to control transmission. To better prepare society for future pandemics, it is important to answer the question of which interventions should be implemented when, where and to which population groups, in order to reduce transmission effectively while limiting societal disruptions due to for example lockdowns. In this study, we examine how Dutch municipalities are connected through workplace-related connections, using unique country-wide registry data of where Dutch people live and where they travel for work. By showing which regions are more or less linked, we provide evidence that can support more targeted control measures, such as regional lockdowns and long-distance travel bans. Our approach could also be extended to other social relationships, e.g., school and family connections, and to other countries, such as Nordic countries where similar registry data are available. Together, our findings provide data-driven evidence on spatial connectivity patterns that can inform the design of targeted control measures as potential alternatives to nationwide mandates in future pandemics.

## 1. Introduction

Respiratory infectious disease outbreaks such as the SARS-CoV-2 pandemic can unfold unevenly across regions [1,2]. This heterogeneity in regional outbreak dynamics is partly due to differences in the degree of spatially structured contacts through which viruses are transmitted across regions [3]. Within the wider population contact networks, colleague networks play a central role in shaping cross-regional population mixing due to their relatively higher volume compared to friend and family links and wider spatial reach compared to classmate and household connections [4].

While some large diary-based studies, such as POLYMOD [5–8] and CoMix [9], quantify colleague connectivity by the frequency and duration of workplace contacts, they do not record the residential or contact locations of the involved individuals. Transmission models based on these surveys either assume homogeneous workplace mixing [10] or at most stratified by age groups [11–13]. The lack of spatial heterogeneity in transmission models leads to overestimation of epidemic growth rate [10,14,15], underestimation of intervention effectiveness [14], and limits the ability of the models to support regional tailored outbreak responses [16–18]. Alternatively, mobility data based on mobile phone signals offer geographical context [3,18,19]. However, mobile phone signals gathered by commercial data providers are subject to uneven regional representativeness due to differential subscription rates and smartphone use across regions.

To bridge this gap, our study leverages large-scale administrative data from Statistics Netherlands (CBS) to quantify the number of employee pairs working for the same company or even local branch for each year from 2019 to 2022. We chose this data source for its population-scale coverage (over 8 million working individuals in the Netherlands) and high-quality and accurate registry (based on standardized payroll tax registrations). The derived data product of geographical connectivity is quantified by counting the undirected colleague pairs indexed by municipality triplets: two residential municipalities indicating where colleagues live and one joint work municipality indicating where they work. We then aggregated these counts at the municipality level to connectivity patterns at two higher administrative units (safety region and province), at which regional pandemic responses can be implemented in the Netherlands [20–22]. Building on these connectivity patterns, we further estimated regional SARS-CoV-2 Omicron onset timing and investigated whether the colleague connectivity was associated with regional onset of Omicron emergence. To showcase the utility of our derived data product for supporting region-tailored interventions, we quantified how a regional lockdown and cross-regional travel ban would reduce total colleague links.

## 2. Results

### 2.1. Geographical colleague connectivity patterns

The number of individuals working in the Netherlands with valid payroll tax registrations ranged from 8.0 million in year 2019 to 8.5 million in 2022. We paired colleagues working for the same company branch and counted undirected colleague links per municipality triplet. The colleague link counts, representing cumulative opportunities for workplace contact, were denoted as $M_{ij}^{k}$, with i and j being the two colleagues’ residential municipalities and k the municipality of their shared workplace. We specifically tracked workplace location because regional pandemic measures can be applied in workplaces without necessarily affecting areas of residence. To reflect that employees in very large companies are unlikely to have contact with all coworkers, we imposed a degree cap, limiting the maximum number of colleagues each individual is allowed to have. With a cap being 100, there were 132 million colleague connections in the year 2021, and a working individual in the Netherlands has, on average, 32 colleague connections. The following results are all for the year 2021 and are based on a cap of 100 colleagues per person.

**Fig 1:**
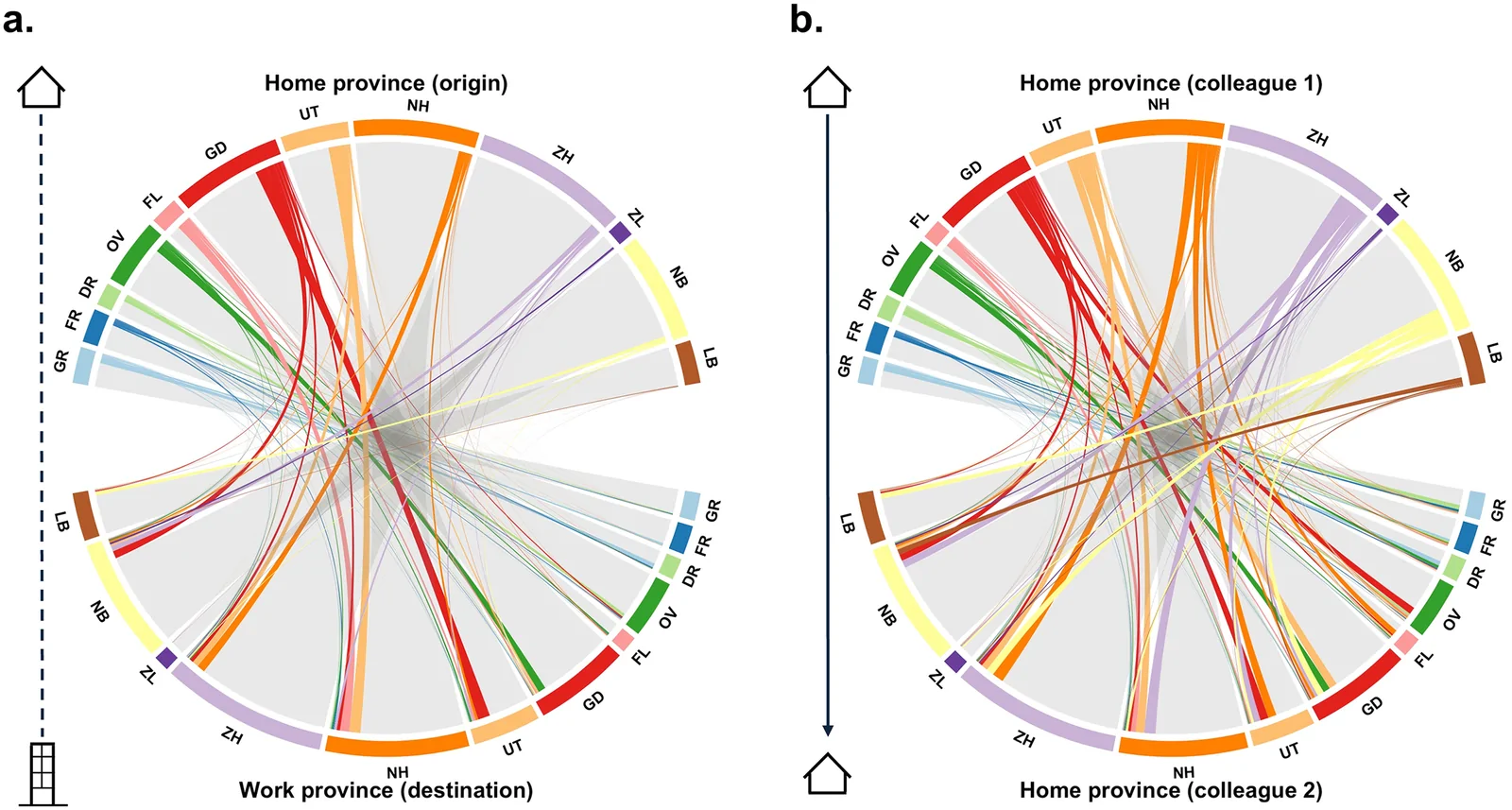
Colleague links between provinces grouped by home-work or home-home pairs. Each panel visualizes the volume of colleague connections (in chords) between pairs of Dutch provinces. Grey ribbons denote within-province connections, while coloured ribbons indicate cross-province links. **(a)** Colleague connectivity between residential and workplace provinces. The top represents the residential province, and the bottom half represents where that individual works, indicating where individuals potentially commute for work. **(b)** Colleague connectivity between two residential provinces. The top half represents the residential province of one colleague (ego), and the bottom half represents the residential province of the colleague working in the same workplace (alter).

Next, we show the extent to which geographical colleague connectivity varied across regions, both in terms of where residents work and where their workplace colleagues live. Fig 1 to Fig 3 visualize the structured patterns of geographical colleague connectivity across the Netherlands, by aggregating colleague counts per municipality triplet ($M_{ij}^{k}$) to higher administrative levels. In Fig 1, we distinguished province-level colleague connectivity in two forms: residence-workplace connectivity (Fig 1a) and residence-residence connectivity (Fig 1b). This distinguishment was available due to the triplet location structure in $M_{ij}^{k}$. In Fig 1a, grey ribbons dominate, indicating that most workers live and work in the same province. Still, coloured ribbons show substantial cross-province commuting. For example, from the perspective of the origin provinces (top half), many Gelderland (GD) residents work in Utrecht (UT), and Utrecht (UT) residents work in Noord-Holland (NH). From the perspective of the destination provinces (bottom half), Gelderland (GD), Utrecht (UT), Noord-Holland (NH), Zuid-Holland (ZH) and Noord-Brabant (NB) are destinations of notably more cross-province colleague connections than other provinces.

Spatial distribution of colleague connections by residential province pairs is visualized in Fig 1b. Similarly, grey ribbons dominate, indicating that most colleagues live in the same province. Even so, we noted non-negligible interprovincial connections shown in coloured ribbons. For instance, many residents of Zuid-Holland (ZH) share workplaces with those from Noord-Holland (NH), Noord-Brabant (NB) and Utrecht (UT). A structural difference between two panels is that the top and bottom margins in Fig 1b have the same totals per province and the same ribbon width per province pair, due to mirror symmetry of the bidirectional home-home connections. However, in Fig 1a, the top (home) and bottom (work) halves have different totals per province and the segments for the same ribbon do not necessarily match in width.

**Fig 2:**
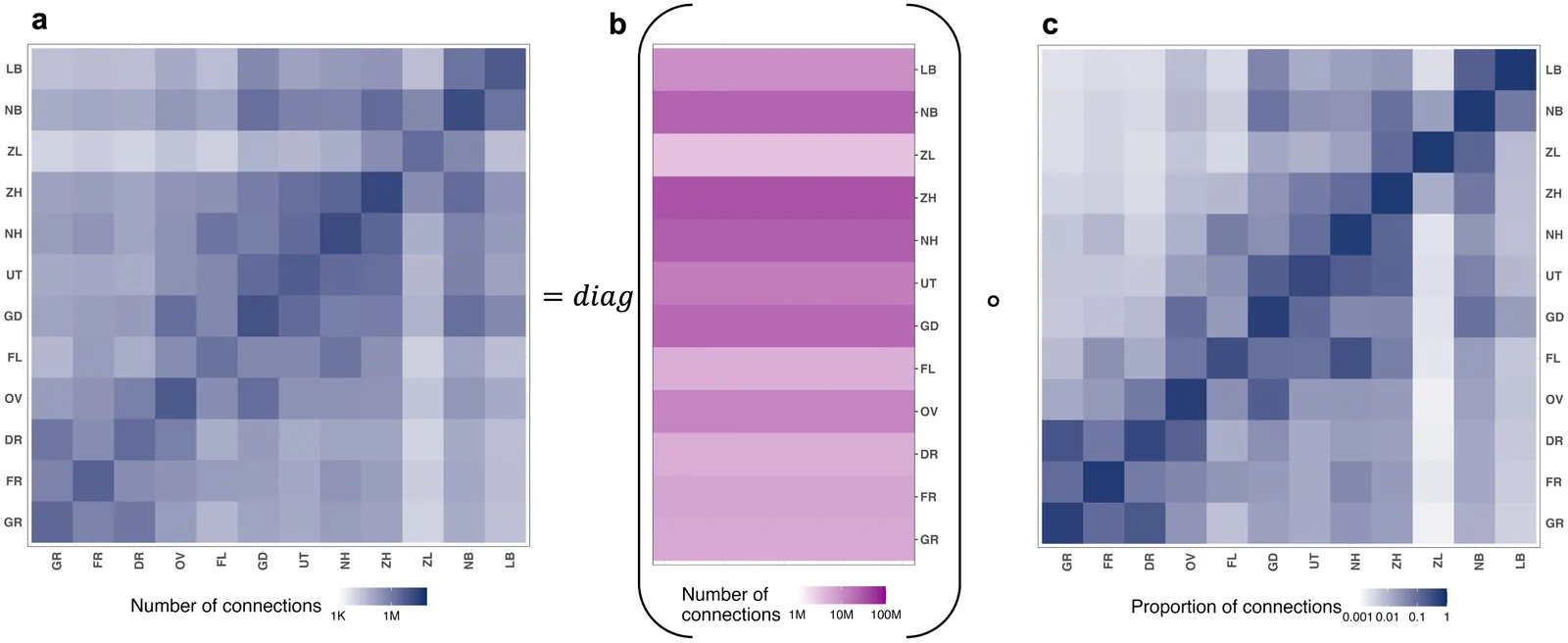
Heterogeneous regional colleague connectivity by residential provinces. **(a)** A matrix with each element being the absolute number of colleague connections between residential province pairs. **(b)** A vector consisting total number of colleague connections per residential province. **(c)** A matrix containing the row-normalized connections between residential province pairs, showing the relative distribution of connections from each province (i.e., the cells in each row add up to one).

Regional variation in residential province connectedness is further visualized in Fig 2 by differentiating total colleague link volume (Fig 2a) from how links are distributed across province pairs after row-normalization (Fig 2c). Fig 2a mirrors the symmetric structure of Fig 1b and shows that most colleague pairs live within the same province. Normalization removes differences largely driven by provincial labour force sizes (as summarized in Fig 2b). For transmission models, Fig 2b serves as an input describing the scale of workplace mixing by province, whereas Fig 2c provides the relative distribution of mixing across provinces. The raw data used to generate this Fig are provided in S1 Table.

**Fig 3:**
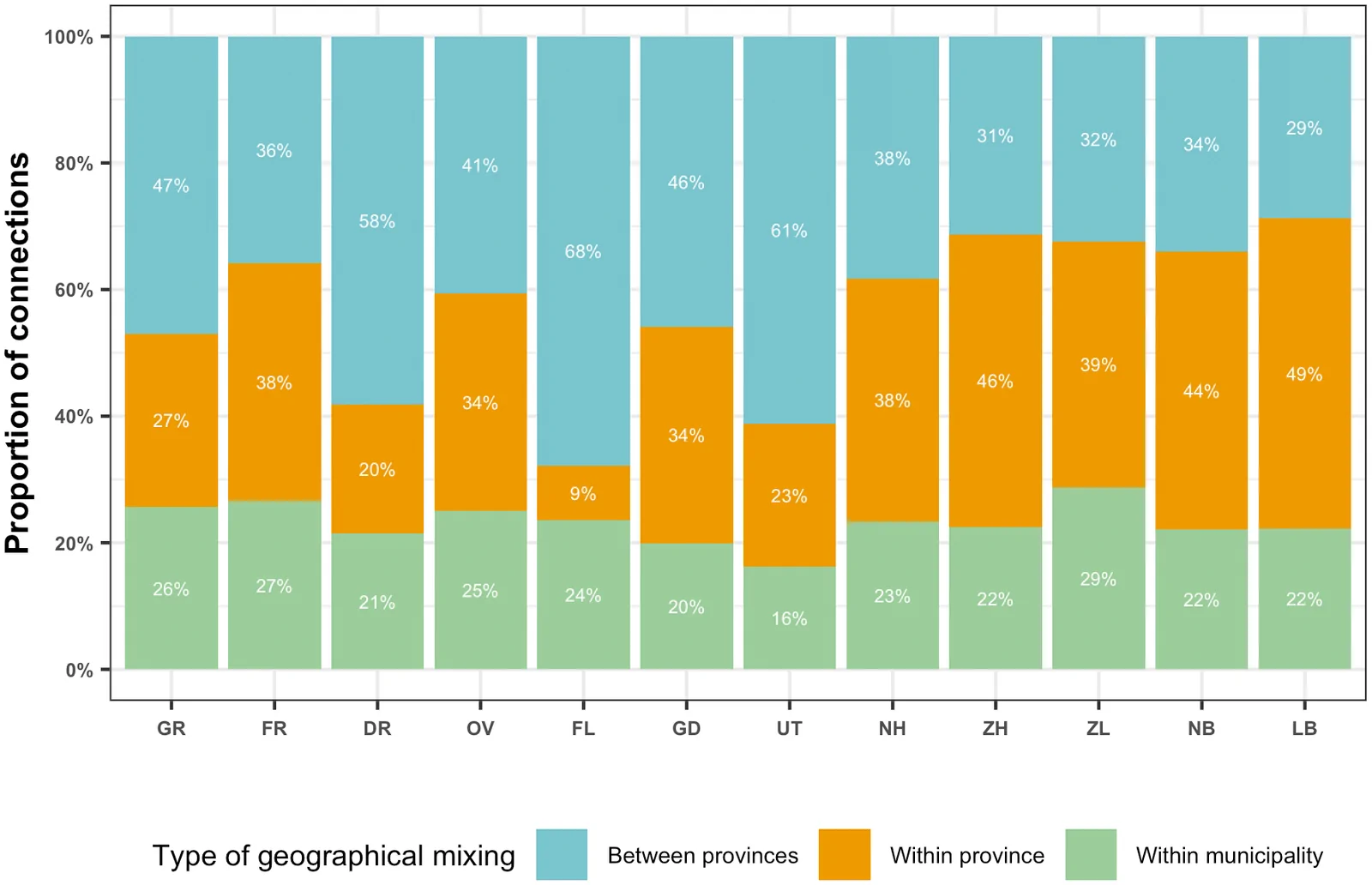
Heterogeneous connectivity shares at different geographic scales. Stacked bar plot shows the proportion of colleague connections originating from individuals living within the same municipality (green), different municipalities within the same province (orange), and between different provinces (blue). Labels indicate the percentage for each connection type per residential province.

Fig 3 subsequently quantifies the heterogeneity of geographical colleague connectivity across administrative scales, reporting for each province, the share of connections of its residents that are within the same municipality (green), between municipalities in the same province (orange), and between provinces (blue). The share of within-municipality connections is similar across provinces, ranging from 16% in Utrecht (UT) to 29% in Zeeland (ZL). In contrast, there is more variation in within-province and between-province connections. Flevoland (FL) has the lowest share of within-province connections (9%) and the highest share of between-province connections (68%), whereas Limburg (LB) shows the opposite pattern, with the highest within-province share (49%) and the lowest between-province share (29%). Utrecht (UT) and Drenthe (DR) also display a high share of inter-province connections, though likely for different reasons: Utrecht (UT) functions as a business hub, attracting commuters for institutions such as national banks, whereas residents of Drenthe (DR) are more likely to travel outside the province for work due to limited local employers. Raw data of this figure were provided in S2 Table.

### 2.2. From connectivity to Omicron onset across provinces

**Provincial Omicron onset timing.** We estimated the onset timing of Omicron emergence per Dutch province, defined as the first date when Omicron incidence exceeded a prespecified case threshold I. We found that the earliest date at which Omicron reached I=30 cases per week differs across the twelve Dutch provinces by almost one month (S3 Fig). Noord-Holland showed the earliest onset, with a mean date of 26^th^ October 2021 and a 95% credible interval from 23^rd^ to 28^th^ October. After Noord-Holland, the next provinces were Gelderland, Zuid-Holland, Utrecht, and Limburg, with average onsets between 7^th^ and 14^th^ November. The virus emerged later in the northern and less populated provinces such as Groningen, Fryslân, Drenthe, and Flevoland, between 20^th^ and 28^th^ November. Uncertainty (shown as horizontal bars) varied, with the widest credible intervals for the least populated provinces Zeeland and Flevoland. Sensitivity analyses of the case threshold I shows that the ranking of onset times is stable across thresholds, with Noord-Holland – home to the country’s largest international airport – consistently estimated as the earliest province to experience the start of the Omicron wave (S4B Fig).

**Fig 4:**
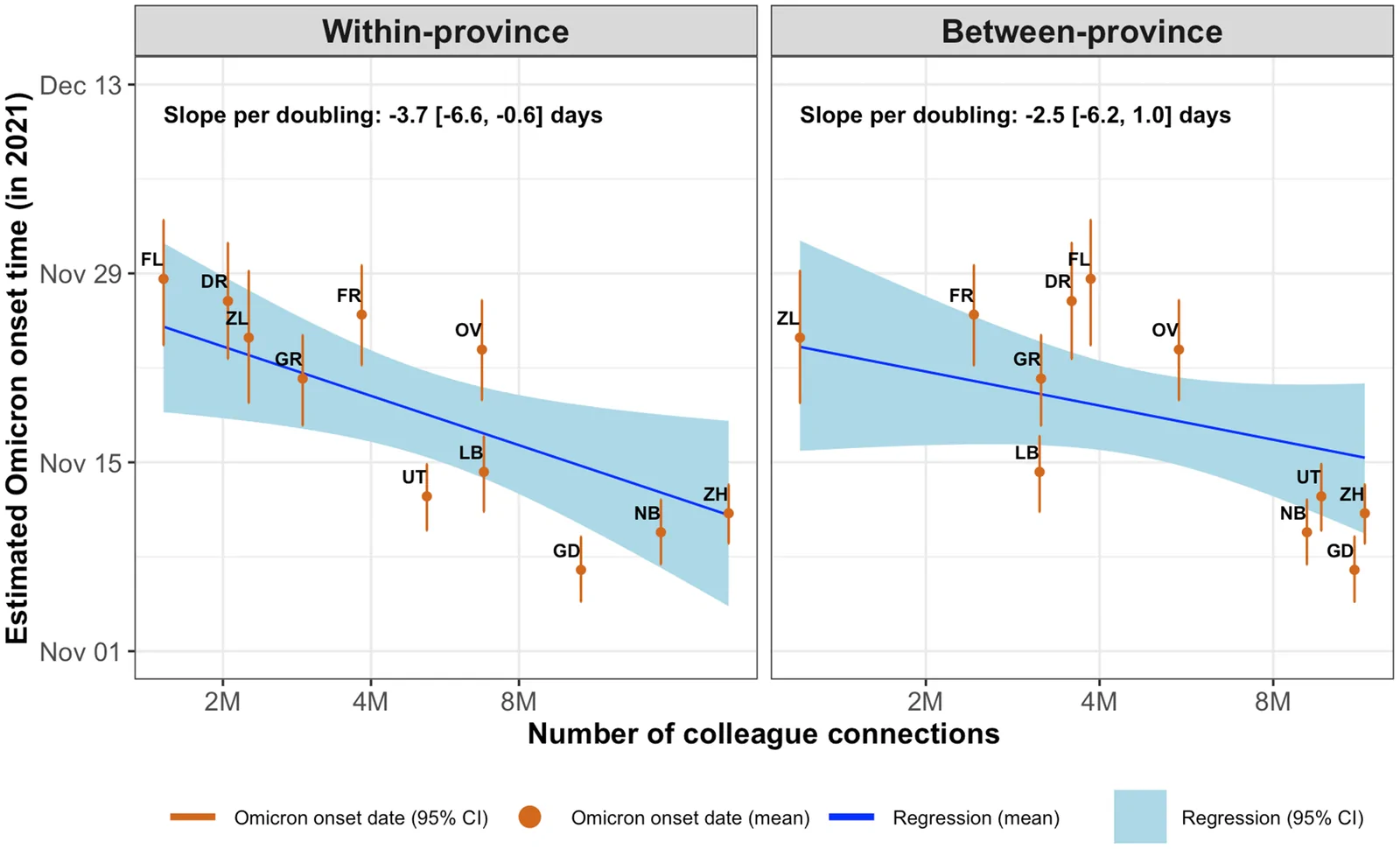
Associations between regional colleague connectivity and Omicron onset timing. Each panel shows estimated Omicron onset time per province (posterior means as circles and 95% credible intervals as error bars) plotted against the number of within- or between-province colleague connections. Blue regression lines and shaded ribbons represent the posterior mean and 95% credible interval from the Bayesian regression inference. The negative trends indicate that provinces with more intra- and inter-province colleague connections are associated with earlier Omicron onset.

**Associations between connectivity and onsets.** Next, we visualized to what extent provincial Omicron onset was associated with colleague connectivity patterns per province except Noord-Holland (NH), where the first few Omicron cases were detected and probably introduced. A two-fold increase in within-province colleague connections was associated with an Omicron onset that was 3.7 days earlier (95% CI: 0.6 to 6.6 days), while a two-fold increase in between-province colleague connections was associated with an onset that was 2.5 days earlier (95% CI: -1.0 to 6.2 days) (Fig 4).

In both panels of Fig 4, some provinces deviate markedly from the overall patterns. For example, Overijssel (OV) had later Omicron emergence than the pattern would suggest. In contrast, Gelderland (GD) experienced an earlier Omicron onset than expected from both the within-province and between-province connectivity regressions.

To investigate the association with case importation from international travel hubs, we also show that colleague connectivity to Noord-Holland (NH), where Amsterdam Airport Schiphol is located, was negatively associated with regional Omicron onset timing (S5 Fig): a two-fold increase in colleague connections with residents of Noord-Holland was associated with an onset that was 2.0 days earlier (95% CI: 0.0 to 4.1 days). Provinces with stronger colleague connections to Noord-Holland (NH), such as Zuid-Holland (ZH) and Utrecht (UT), tend to have earlier onsets, while the virus emerged later in more distant provinces like Zeeland (ZL).

### 2.3. Implications for regional interventions

After deriving the spatial workplace connectivity patterns, we now show how these patterns can be used to inform region-targeted control measures. In the following analysis, we quantified the number and proportion of removed workplace links under two regional intervention scenarios: (1) regional lockdowns, where workplace links are removed when employees either work or live in the targeted region, and (2) cross-regional travel bans, where links are removed when residents of the targeted region worked in another region, or residents of another region worked in this targeted region. A distinction between the two intervention scenarios was that under cross-regional travel bans, workplace links where both employees live and work in the targeted region would not be affected.

**Fig 5:**
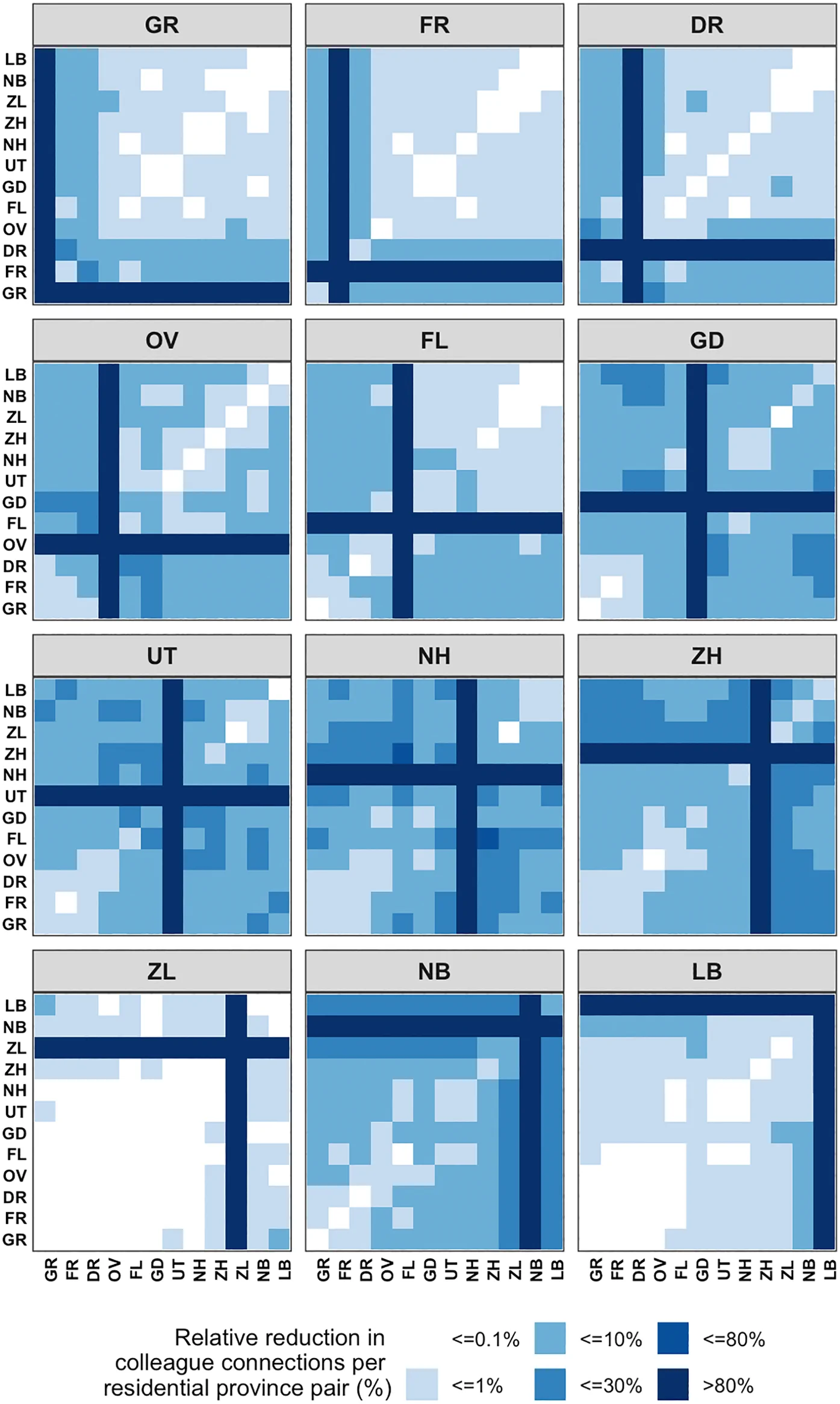
The reductions in colleague links per residential province pair under province-level lockdowns. Each panel visualizes the relative reduction in colleague connection between residential province pairs when the province labelled at the top of the panel is placed under lockdown. The darker the shades, the larger proportions of connections would be removed. Values were derived from Fig 2a.

For the regional lockdown scenario, each panel in Fig 5 shows the relative reductions of colleague connections per residential province pair when one province was placed under lockdown. The panels are symmetric because removed links between residents of province A and province B were counted the same as links between residents of province B and province A, the same structure of Fig 2a. Colleague connections of people living in the locked province were entirely removed, clearly shown by the darkest shades in all panels. Off-diagonal shading reflects the reduction in connections between colleagues that both live in a province that is not locked down and are linked through a workplace in the locked province. Lockdowns in Noord-Holland (NH), Zuid-Holland (ZH), and Utrecht (UT) affect the largest number of cross-province province pairs (dark off-diagonal cells), consistent with dense interprovincial connectivity centred on the Netherlands’ western urban corridor (Randstad) around the country’s four largest cities. Notably, Gelderland (GD) and Noord-Brabant (NB) also exert national influence in connectedness patterns. The municipalities with the largest colleague-link removal were mainly large residential and employment centers, with rankings differing between the two intervention types (S4 Table). Analogous maps for safety-region lockdowns are provided in S6 Fig which demonstrate the heterogeneous impact of locking down one of the 25 Dutch safety regions.

**Fig 6:**
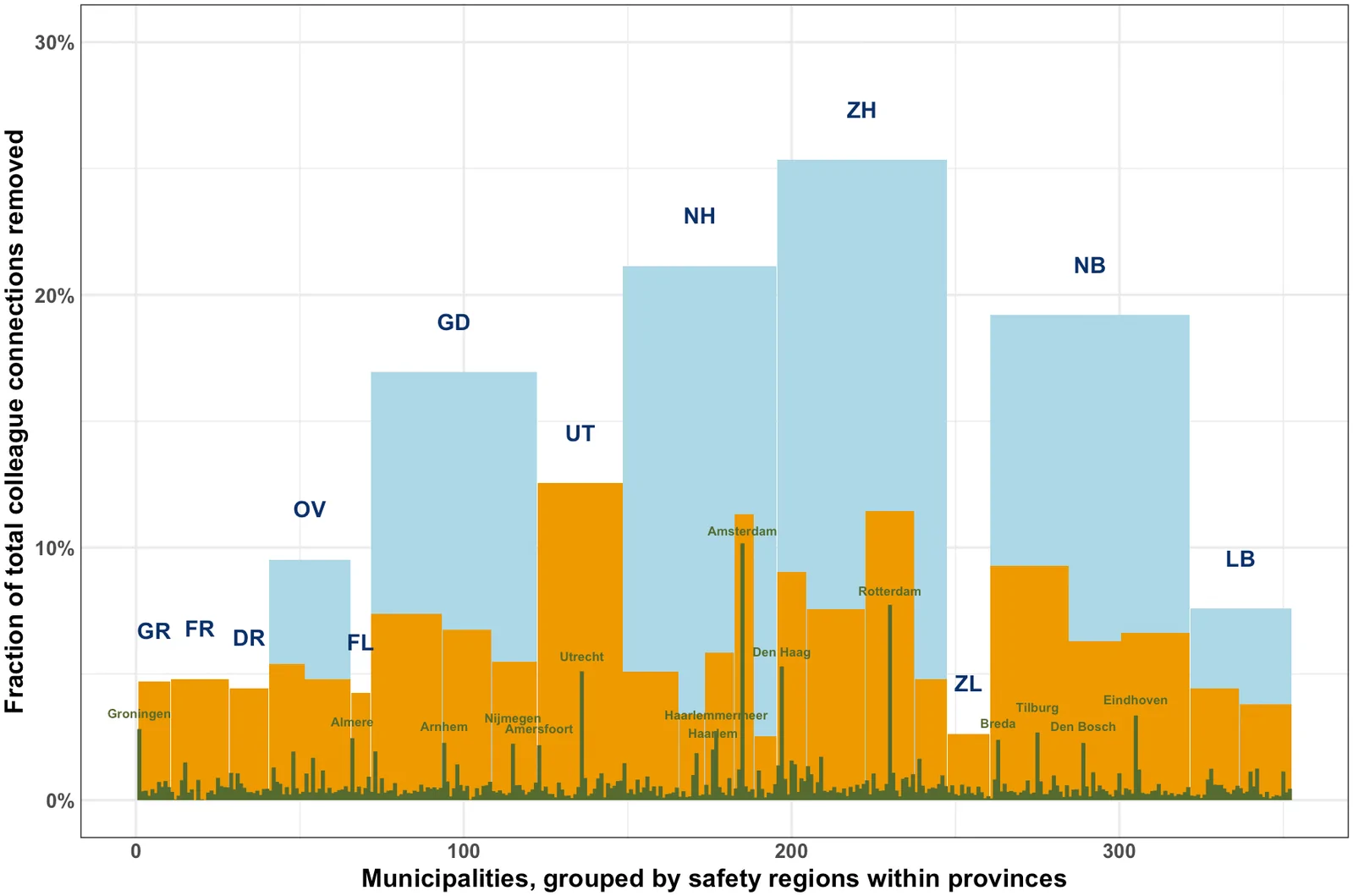
The relative reductions in total national colleague links per residential region under province-level lockdowns. Each bar shows the share of all colleague connections that would be removed by locking down a single administrative unit at one of three levels: municipality (green), safety region (orange), or province (blue). The 352 municipalities are grouped by 25 safety regions within 12 provinces, using standard numbering from north to south. Province abbreviations are labelled in blue, while the municipalities with 15 largest contributions are labelled in green.

After aggregating the removed connections to the national level, the fraction of total colleague connections removed under a regional lockdown was unevenly distributed across regions (Fig 6). Locking down one of the twelve provinces (blue bars) would remove only 2.6% (province ZL) to as much as 25.3% (province ZH) of all colleague connections. The range when safety regions were placed in lockdown was 2.5% to 12.5% (orange bars). When locking down municipalities, the proportion of removed workplace links is highly skewed (green bars). Locking down Amsterdam alone would remove 10.0% of all national colleague connections, while there are 305 municipalities each contributing less than 1% of all colleague connections. Municipalities with the top 15 contributions to colleague connections are labelled in green.

Though regional labour force size is one of the main drivers for a region’s contribution to national total colleague links, we successfully identified business hubs (where there are not necessarily many residents but a high concentration of companies) by including workplace municipality as a third spatial attribute. For instance, though municipality Haarlemmermeer has about 25% less working population compared to municipality Almere (88.9 thousand vs. 119.1 thousand), locking down Haarlemmermeer would remove more colleague connections than Almere (2.7% vs. 2.4%).

**Fig 7:**
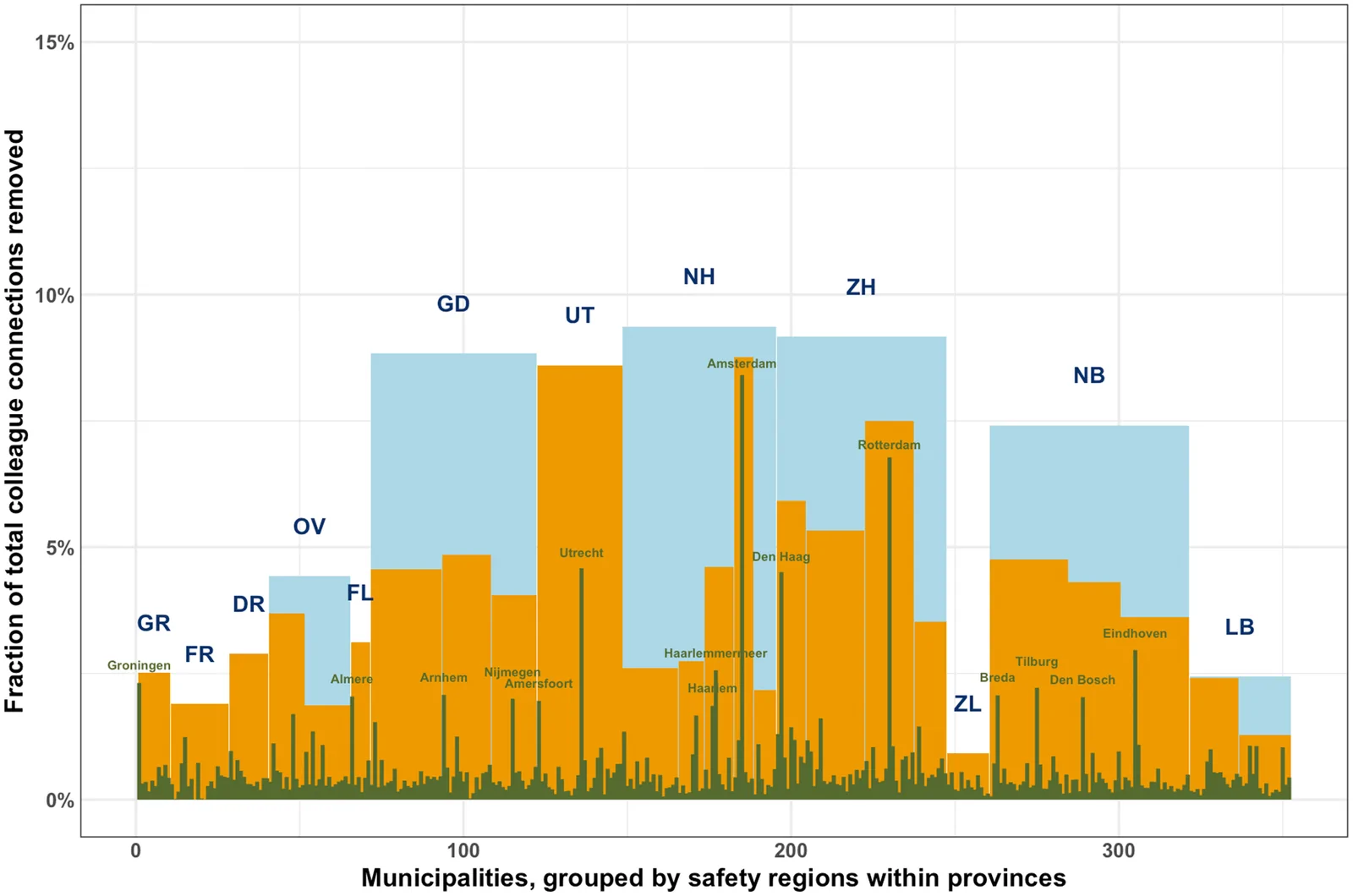
The relative reductions in total national colleague links per residential region under cross-regional travel bans. Each bar shows the share of all colleague connections that would be removed due to crossing the boundary of a single administrative unit (i.e., commuting into or out of that region). The rest of the captions are the same as Fig 6.

Cross-regional travel bans were an alternative regional intervention to the uniform regional lockdown, by removing only links that cross regional boundaries while preserving within-region colleague links. Under cross-region travel bans, the overall reductions in total colleague links (up to 9.4% as shown in Fig 7) were consistently lower compared to regional lockdowns (up to 25.3% as shown in Fig 6). Labour force size explained less of the reduction under travel bans. S7 Fig shows that highly populated regions are not necessarily the most influential under cross-regional travel bans. For example, province Zuid-Holland (ZH) has about 25% larger labour force than Noord-Holland (NH) (2.0 million vs. 1.6 million), but imposing a travel ban in ZH removes fewer connections than a ban in NH (9.1% vs. 9.4%), which was not the case for regional lockdowns (25.3% vs. 21.1%).

### 2.4. Sensitivity analysis

The spatial connectivity patterns show broadly consistent province-level contrasts across different choices of the degree cap D (i.e., the maximum number of colleague connections an individual is allowed to have), although the total inferred colleague links in the 2022 sensitivity analysis (ranged from 61 million to 239 million) and their spatial compositions changed with the choice of cap (S8 Fig). As the cap increased from 50 to 200, the percentage of between-province connections rose by up to 5.56%, at the expense of within-municipality links. These patterns were also robust to the arbitrary choice of random seed used to assign employees to local branches. Across 10 different random seeds, the province-level shares of within-municipality, within-province, and between-province colleague connections (same metric as presented in Fig 3) varied by at most 0.10 percentage points. And the largest relative difference across the 10 seeds for any single entry of the two-dimensional mixing matrix (same metric as presented in Fig 2a) was 1.99%.

The ranking of provincial Omicron onset timing was stable across different choices of $e_{\text{min}}$ (i.e., beyond which weekly incident cases defined the start of loglinear model, S4A Fig) and to the threshold for onset I (i.e., beyond which weekly incident cases defined the emergence of Omicron, S4B Fig).

## 3. Discussion

Our study presents a generalizable framework to translate individual-level registry data into spatial connectedness patterns, by pairing individuals working in the same local branch. We derived a highly fine-grained and broadly reusable data product which quantifies geographical connectedness of colleague networks within and between Dutch municipalities. It is reassuring that our hypothesis of colleague connectivity being relevant to spatial outbreak dynamics is supported by the association between connectivity patterns and regional SARS-CoV-2 Omicron onset timing. We further quantified reductions in colleague connections under two regional interventions. These reductions can serve as inputs for adjusting spatial mixing matrices in future transmission models which aim to quantify the epidemiological impact of regionally tailored interventions. The reduction in colleague connections under simultaneous lockdowns in two or more provinces can be quantified using the same workflow.

Our geographical colleague connectedness corroborates the assumptions in recent studies [14,16] that population mixing differs by geographic scale within a country. However, the share of region-specific mixing in these studies was assumed to be fixed and uniform across provinces, whereas our estimates show substantial interprovincial variations.

As for the provincial Omicron onset timings, Noord-Holland (NH) was estimated to experience the earliest Omicron onset regardless of different case thresholds. This clearly reflects elevated importation risk from international flights arriving at Amsterdam Airport Schiphol. Geographical colleague connectivity patterns are correlated with part of the variation in provincial Omicron onset, though other factors are likely to contribute as well. For example, even though Flevoland (FL) was highly connected to Noord-Holland (NH), Omicron emerged much later than the overall pattern (S5 Fig). This might be explained by the first association analysis, where Flevoland (FL) has relatively fewer within-province colleague connections (Fig 4a). Overijssel also had a late Omicron emergence, but instead of lacking within- or between-province colleague links, an alternative mechanism might be that our association models do not capture whether each region is linked to the region of higher or lower transmission risks. In contrast, Gelderland (GD) experienced a relatively early estimated Omicron onset. A plausible explanation might be the substantial student mixing surrounding its university hubs such as Wageningen University and Radboud University.

We noted that the fraction of colleague links removed under a regional lockdown is related to the labour force size per administrative unit, as regions with more workers generally contribute more to the national total workplace links. However, workforce size alone does not determine the volume of inferred workplace links, but also factors such as the number and size distribution of workplaces. Moreover, the number of workplace links does not shape transmission risks alone; the latter also depends on transmission-related characteristics such as in-person contact intensity and remote-work patterns.

We also highlighted that by including workplace locations in the spatial connectedness patterns, we were able to identify regions that were hubs to businesses, but not necessarily hubs to residents. As such, our data product supports a better design and evaluation of subnational approaches compared to earlier work [18], by allowing a regional intervention to target workplace locations without necessarily affecting residence-areas. More importantly, when infection levels are high in a specific area, regions that are strongly connected to it through workplace links may represent plausible candidates for enhanced surveillance or early-warning prioritisation. In this context, mobility-restricting interventions, such as regional lockdowns or cross-regional travel bans, may disrupt workplace links and thereby reduce potential workplace-related pathways through which infections could be seeded and spread onward to other regions.

We further argue that the proposed framework for translating microdata to spatial connectivity patterns can be generalized to other population contact settings or transferred beyond the Netherlands. The key prerequisite is access to individual-level registry data that include residential locations, linkages to relevant entities such as workplaces or schools and their geographic locations, and last but not least, legal and ethical access to the data. For example, apart from registrations on colleagues and companies, CBS also links students with their schoolmates, residents within the same households, and with their family members. There is ongoing work by our research team to characterize geographical connection patterns for student, family and household networks in the Netherlands, and investigate effectiveness of regional pandemic responses accordingly. For generalizability to international contexts, administrative data in the Nordic countries are systematically collected and have great potential to be linked, including microdata from Statistics Norway [23], microdata from Statistics Denmark [24,25] and microdata from Statistics Sweden [26]. With harmonized geographic units, standardized identifiers for individuals and entities, and our proposed framework, it is feasible to construct spatial connectivity for selected network layers in these countries at comparable municipal or aggregated geographical scales. Given that Norway and Sweden are 8 and 11 times larger in land area compared to the Netherlands, we expect stronger within-municipality social network connectivity, with fewer and more spatially fragmented long-distance connections. And a recent study from Denmark, where there is similar land area and cross-regional commuting tendencies, inferred plausible transmission clusters by linking registry social network data with sequencing data [27], showing great potential of administrative data outside the Netherlands.

Our study has five limitations, for which we describe the consequences and planned mitigation steps below. First, in our two-stage Bayesian framework for estimating provincial Omicron onset, we used a plug-in approach by multiplying the posterior Omicron proportions by the total weekly COVID-19 cases and used this product as the rate parameter in the subsequent Poisson model. By doing so, only parameter uncertainty from the first stage is propagated to the second stage, while data uncertainty (i.e., the binomial sampling variability in Omicron cases) is omitted. In other words, the associations might be more uncertain if we had considered data uncertainty. Second, colleague connections in the context of this study do not imply real-world close contacts, due to lack of information on the frequency and duration of physical contacts per connection. Challenges remain when combining connections from different network layers as an average household contact is more likely to be more intimate and longer in period compared to a workplace contact. This gap could be partly addressed in future modelling work by incorporating insights on the relative weights of different types of social network connections from previous contact surveys. Third, under the near-even split assumption, we assumed that different branches of the same company within the same municipality have approximately equal numbers of employees. In the absence of the degree cap, assigning the same total number of employees to the same number of local branches evenly resulted in an underestimated total colleague links compared to the more skewed branch size distribution. However, with a cap on the maximum number of connections per person, this bias was reversed for companies where the average number of employees per branch was around this cap value.

Fourth, current colleague connectivity has not explicitly included granularity on industrial sectors, occupations, types of contracts, or remote-work potential, therefore limiting the actionability of potential intervention strategies such as targeting on certain sectors. We could stratify the connectivity patterns to different sectors or contract types, but another challenge would be to fulfil the CBS output guideline where at least 10 observations are required to avoid group disclosure risks [28]. Still, future work can focus on predefined subsets of sectors or contract types, thereby avoiding the need for fine-grained breakdowns across all sectors while still enabling more informative analyses within allowed disclosure limits. Fifth, it is important to note that the potential impact of regional interventions is based solely on proportion of (long-distance) colleague connections being removed. Future work is needed to integrate several different social network layers such as classmates and families. It should also quantify the epidemiological impact of regional interventions by incorporating these spatial connectivity patterns into transmission modelling frameworks.

In conclusion, we establish a reusable data product of geographical connectedness in colleague networks. This workplace connectivity provides a valuable spatial input for future transmission modelling and policy evaluation, particularly for representing how workplace links are distributed within and between regions. The Omicron analysis reassures that how such connectivity patterns are associated with regional differences in outbreak timing; further dynamic transmission modelling is needed to assess the impact of interventions that affect colleague connectivity. Also, we conclude that our workflow of inferring colleague networks and spatial connectivity patterns from individual-level registry data is valuable in its generalizability to other population contact settings and international contexts beyond the Netherlands.

## 4. Materials and methods

### 4.1. Ethics statement

This study used registry data on individuals and companies from the Statistics Netherlands (CBS). Formal ethical approval for the use and publication of these data in the context of the PDPC-SURE Frontrunner project was obtained from the Erasmus MC Medical Ethical Council, under registration number MEC-2025-0338. Additionally, the research question and the required datasets were reviewed by CBS for feasibility and compliance with the General Data Protection Regulation (GDPR). Data access was provided exclusively through the secure CBS Microdata environment. All outputs from registry data were checked by CBS through a disclosure risk assessment before being released from the secure environment.

### 4.2. Overview of the methodology

**Fig 8:**
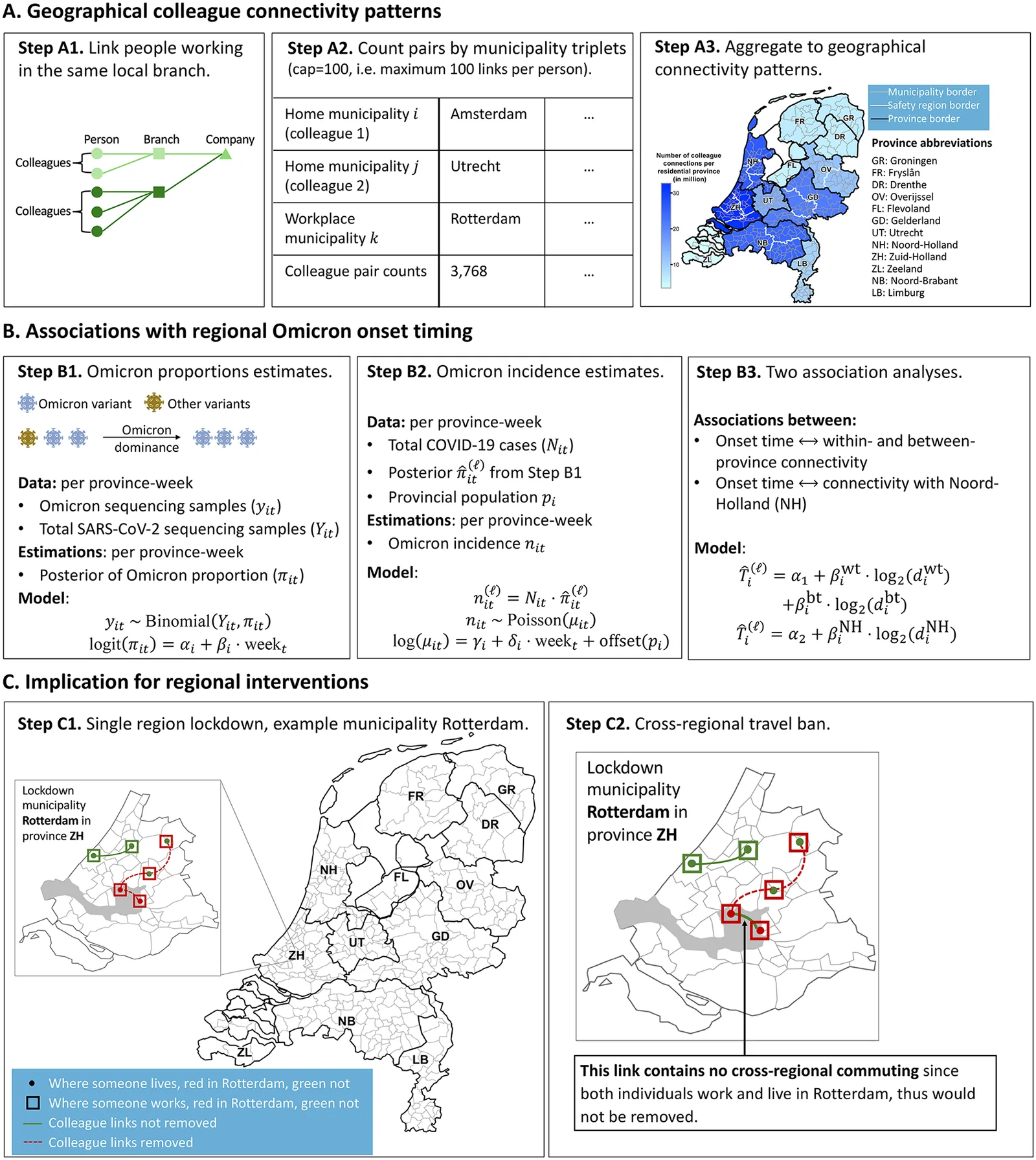
Overview of the data and methods. Our analysis framework consists of three steps: (A) construction of geographical colleague connectivity patterns across the Netherlands for its 352 municipalities, 25 safety regions, and 12 provinces; (B) estimation of provincial onset timing of SARS-CoV-2 Omicron variant, and evaluation of how this onset is associated with regional connectivity patterns; and (C) quantification of the impact of regional interventions on colleague connections within and between regions. The municipal and provincial boundaries shown in panels (A) and (C) were plotted using boundary data retrieved in R via the cbsodataR package, which sources the official boundary statistics from CBS [33].

This study integrates administrative data and epidemiological surveillance data to investigate the role of geographical connectivity in shaping subnational transmission dynamics during the Omicron wave in the Netherlands. Fig 8 provides an overview of the multi-step workflow and data sources which are discussed in the following sections. First, we paired employees working for the same company by using employment relationships recorded in Dutch payroll tax registers and accessed via Statistics Netherlands (CBS) Microdata environment (Step A in Fig 8), following a similar methodology as Van der Laan et al. (2023) [29]. For large companies with local chain stores, such as supermarkets, people working at different branches are unlikely to have regular workplace contacts. Thus, we further refined this network by identifying employees of the same parent company who most likely work at the same local branch, using branch counts per branch location. Details of branch assignment algorithm can be found in S1 Text. Then we linked individuals’ residential municipalities to the municipality where they work, to quantify the number of work-related connections per municipality triplet (two home municipalities for two individuals and their shared working municipality). These links identified by shared workplace represented cumulative opportunities for contact, rather than transmission-relevant contacts capturing the frequency or duration of in-person interactions. The datasets with municipality-level geographical colleague connectivity are open access [30] together with the code for reproduction within CBS Microdata environment.

Second, we estimated the onset timing of the SARS-CoV-2 Omicron variant per province (Step B in Fig 8) by combining province-level case data from the National Institute for Public Health and the Environment (RIVM) and variant-specific sequencing data from the Global Initiative on Sharing All Influenza Data (GISAID) [31,32]. Then we quantified the associations between regional colleague connectivity and Omicron onset timing for each province. By doing so, we evaluated whether provinces with higher intra- or inter-province connectivity were associated with earlier onsets, and whether the time lag in onset relative to the first affected province was associated with the level of connectivity to that province.

Third and last (shown as Step C in Fig 8), we quantified the removed workplace links under regional interventions. Two regional interventions were implemented: (1) regional lockdowns, which would remove all colleague links that involve individuals living or working in the locked region; (2) cross-regional travel bans, which would remove all links that involve individuals commuting into or out of the targeted region.

### 4.3. Construction of geographical colleague connectivity patterns (Step A)

Here, we describe the algorithm and underlying assumptions to count the number of colleague links per municipality triplet $M_{k}^{i,j}$, where i and j are the residential municipalities of two colleagues and k is their shared work municipality. These counts were then aggregated into metrics and matrices that characterize geographical patterns of colleague connectivity. First, individual-level administrative records were linked to group people employed by the same company. For companies with multiple branches within the same municipality, where the data did not specify the branch in which an employee worked, individuals were randomly assigned to branches while ensuring approximately equal branch sizes. Second, colleague links were counted for each branch and municipality triplet, aggregated across branches, and weighted by an assumed maximum number (cap) of colleagues per individual, with sensitivity analysis for this cap. Finally, links per municipality triplet were aggregated to summarize the spatial patterns in colleague connectivity across the Netherlands.

**Registry data.** This study uses person- and company-level registry data [34] from Statistics Netherlands (CBS) covering the years 2019–2022, accessed via the secure CBS Microdata environment under approved data use agreements. Across the period of 2019–2022, these individual-level registry data cover 8.0 million (year 2019) to 8.5 million (year 2022) working individuals and 8.5 million (year 2019) to 9.0 million (year 2022) work income statements annually. Details of what these data entail and how these data were linked can be found in S1 Text.

**Link people working in the same branch (Step A1).** For companies that operate more than one branch within the same municipality, the registry data do not identify which local branch an individual is linked to. To avoid inflating colleague links by connecting individuals not co-located, we developed a branch-assignment algorithm to allocate each employee to the most probable branch and grouped colleagues accordingly, as follows. Let N be the number of employees working in the same municipality for a given company, and B the number of local branches in that municipality. Then the following three cases can be considered:

(1) If B=1, all N employees work at the same branch, so $\frac{N(N-\text{1})}{\text{2}}$ undirected colleague links in total.
(2) If B>1, N employees are deterministically and near-evenly allocated across B branches within the municipality. With near-even split, branch size $N_{B}\;$is either rounding down to the nearest integer $\left\lfloor \frac{N}{B}\right\rfloor$ or rounding up to the nearest integer $\left\lceil \frac{N}{B}\right\rceil$, chosen such that the sizes sum exactly to the N. Within-branch colleague pairs can be computed accordingly (explained in equation 1).
(3) If there is no information on B, we assume B=1.

**Count pairs by municipality triplet (Step A2).** After identifying co-workers working in the same local branch, individuals were clustered based on their home municipalities. Consider a branch b located in municipality k with $N_{b,k}$ employees. Let $N_{b,k}^{i}$ and $N_{b,k}^{j}$ be the number of those employees who live in municipality i and j, respectively. Using undirected counting, unique colleague pairs within branch b for triplet (i,j,k) are represented as:


$$\begin{array}{c} \begin{array}{c} M_{b,\;k}^{i,j}=\left\{ \begin{array}{c} N_{b,k}^{i}\cdot N_{b,k}^{j},\;\text{if}\;i\text{≠}j\\ \frac{N_{b,k}^{i}\cdot \left(N_{b,k}^{i}-\text{1}\right)}{\text{2}},\;\text{if}\;i=j \end{array}\right.\; \end{array} \end{array}$$ (1)

The symmetry $M_{b,\;k}^{i,j}=M_{b,\;k}^{j,i}$ holds as pairs are undirected. Aggregating over all branches located in municipality k gives the total number of colleague pairs for triplet (i,j,k):


$$\begin{array}{c} M_{k}^{i,j}=\sum_{b\;\text{locates in}\;k}M_{b,k}^{i,j} \end{array}$$ (2)

**Degree cap.** In large workplaces, individuals are expected to interact with only a subset of other employees working in the same branch office. We therefore let each employee have a degree cap D as the maximum number of colleague connections per individual is allowed to have over the employment period. We followed the decision of Statistics Netherlands (CBS) and set as default: D=100 [29]. This order of magnitude aligns with Dunbar’s theory of a cognitive limit on human group size being 148 as having stable relationships [35]. While this cap exceeds the average of 13.4 contacts per individual per day reported in diary-based contact studies [5], it accounts for potential cumulative contact opportunities over longer time periods.

Subsequently, for each branch b with $N_{b}\;$employees, we computed a weight based on the cap to scale the count of colleague pairs within branch b per municipality triplet:


$$\begin{array}{c} \begin{array}{c} w_{b}=\left\{ \begin{array}{c} @c\frac{D\cdot (D-\text{1})}{\text{2}M_{b,\;k}^{i,j}},\;\text{if}\;N_{b}>D\\ \text{1},\;\text{if}\;N_{b}\leq D \end{array}\right.\; \end{array} \end{array}$$ (3)

The total number of colleague pairs in equation (2) is weighted accordingly:


$$\begin{array}{c} {\tilde{M}}_{k}^{i,j}=\sum_{b\;\text{locates in}\;k}w_{b}\cdot M_{b,k}^{i,j} \end{array}$$ (2′)

S1 Text documented the details of how to assign colleagues to the most probable local branches and thereby count 131,910,029 colleague connections in total.

**Aggregate to geographical connectivity patterns (Step A3).** In the Netherlands, municipalities, safety regions and provinces are the three regional administrative levels at which regional responses can be implemented [20,21]. We aggregated ${\tilde{M}}_{k}^{i,j}$, the number of colleague pairs per municipality triplet, to the safety region and province scales. The following summary statistics were derived to capture both the volume and share of colleague connections: (1) count of colleague connections by home-work or home-home province pairs; (2) a connection matrix by residential province pair with absolute connection counts, together with its row-normalized version to remove population-size scaling effects; (3) the share of connections that are within municipality, within province and between provinces.

### 4.4. Associations with regional Omicron onset timing (Step B)

In this section, we describe the data and framework to estimate the timing of Omicron emergence across Dutch provinces and relate this with geographical colleague connectivity patterns.

**Sequencing and incidence data.** We used two publicly available epidemiological datasets to estimate the timing of Omicron emergence per Dutch province. First, SARS-CoV-2 genome sequencing samples were downloaded from GISAID, a global data-sharing initiative for SARS-CoV-2 genomes and metadata [32]. These sequences were collected as part of the Dutch national germ surveillance program, in which Dutch laboratories mapped all the building blocks of the virus’ RNA to monitor variant prevalence and detect emerging variants of concern [36]. A total of 165,219 SARS-CoV-2 sequences collected from the 12 Dutch provinces were downloaded from GISAID, spanning from February 9^th^, 2020 to March 31^st^, 2023. By restricting the date starting from the week beginning December 28^th^, 2020, the first date when all 12 provinces had available genome sequences, until the week ending with March 31^st^, the end of sequence collection in the Netherlands, 157,891 sequences were left for analysis. To mitigate reporting fluctuations within a week, data were aggregated at the weekly level.

Second, municipality-level COVID-19 incidence was retrieved from RIVM repository [36], spanning from February 28, 2020, to March 31, 2023, the date when public testing facilities were closed. Data were aggregated to weekly new cases per province to avoid artificial fluctuations seen in daily case reporting and to align the time gap between case testing and sequencing.

**Omicron proportions estimates (Step B1).** We then quantified provincial heterogeneity in the onset timing of Omicron emergence, defined as the first date when Omicron-positive weekly cases surpassed a pre-defined threshold. As an emerging variant after the Delta wave, Omicron exhibited substantial immune evasions which resulted in infections of individuals regardless of vaccination or infection with earlier variants [37,38]. The immune evasions of Omicron reduced bias due to varying immunity levels across provinces. Moreover, compared to earlier SARS-CoV-2 variants, Omicron emerged during a period when large-scale sequencing and testing infrastructure were in place in the Netherlands [36]. The higher surveillance capacity led to more complete and consistent data coverage across provinces, improving the credibility of Omicron onset timing estimates.

We developed a two-stage Bayesian inference workflow to simulate weekly Omicron cases over time. A hierarchical logistic regression model was first fitted to sequencing data. We modelled the number of Omicron-positive sequences $y_{it}$ among all sequenced cases $Y_{it}$ from province i in week t using a binomial likelihood,


$$\begin{array}{c} \begin{array}{c} y_{it}\;\sim \;\text{Binomial}\left(Y_{it},\;\pi_{it}\right) \end{array} \end{array}$$ (4)

where $\pi_{it}$ is the probability that a sequenced case was Omicron-positive. The log-odds of $\pi_{it}$ were modeled with a linear predictor as:


$$\begin{array}{c} \begin{array}{c} \text{logit}\left(\pi_{it}\right)=\alpha_{i}+\beta_{i}\cdot {\text{week}}_{t} \end{array} \end{array}$$ (5)

with $\alpha_{i}$ and $\beta_{i}$ being province-specific intercepts and slopes, respectively. These parameters were given hierarchical priors, so that:


$$\begin{array}{c} \alpha_{i}\;\sim \;N\left(\mu_{\alpha },\;\sigma_{\alpha }\right),\;\beta_{i}\;\sim \;N\left(\mu_{\beta },\;\sigma_{\beta }\right) \end{array}$$

where hyperparameters $\left(\mu_{\alpha },\;\mu_{\beta },\;\sigma_{\alpha },\;\sigma_{\beta }\right)$ had vague priors specified in S3 Table. The model was fitted in Stan using Hamiltonian Monte Carlo (HMC) via the cmdstanr package (version 0.9.0). We ran 4 chains, each with 400 warm-up iterations followed by 400 sampling iterations.

**Omicron incidence estimates (Step B2).** The weekly cases of Omicron $n_{it}\;$in province i, week t, was assumed to grow exponentially during the early phase, which is defined as the four-week period starting from the first week when weekly Omicron incidence exceeded a prespecified threshold $e_{\text{min}}$, and continuing with an increase in the subsequent week’s incidence. The four-week window was chosen based on Grabowski et al. (2022) [39].

Because $n_{it}$ was unobserved, we imputed the expected values by multiplying the reported total COVID-19 new cases per week, $N_{it}$, with the 1,600 posterior draws of the Omicron proportion ${\hat{\pi }}_{it}^{\left(\text{𝓁}\right)}\;$ from the logistic model,


$$\begin{array}{c} \begin{array}{c} n_{it}^{\left(\text{𝓁}\right)}=N_{it}\cdot {\hat{\pi }}_{it}^{\left(\text{𝓁}\right)},\text{ 𝓁}=\text{1},\;\text{2},\;\ldots ,\;\text{1}\text{6}\text{0}\text{0} \end{array} \end{array}$$ (6)

We then modelled the province-level weekly Omicron cases $n_{it}$ using a Poisson generalized linear mixed model with a log link,


$$\begin{array}{c} \begin{array}{c} n_{it}\;\sim \;\text{Poisson}\left(\mu_{it}\right) \end{array} \end{array}$$ (7)

$$\begin{array}{c} \begin{array}{c} \text{log}\left(\mu_{it}\right)=\gamma_{i}+\delta_{i}\cdot {\text{week}}_{t}+\text{log}\left(\frac{p_{i}}{\text{1}\text{0}\text{0},\text{0}\text{0}\text{0}}\right) \end{array} \end{array}$$ (8)

where $p_{i}$ is provincial population size included as an offset, and province-specific intercepts $\gamma_{i}\;$and slopes $\delta_{i}$ were modeled hierarchically,


$$\begin{array}{c} \begin{array}{c} \gamma_{i}\;\sim \;N\left(\mu_{\gamma },\;\sigma_{\gamma }\right),\;\delta_{i}\;\sim \;N\left(\mu_{\delta },\;\sigma_{\delta }\right) \end{array} \end{array}$$

Each simulated series $n_{it}^{\left(\text{𝓁}\right)}$ was then used as input to the model in equation (8), thereby propagating sequencing uncertainties to Omicron case estimates. We used a Poisson likelihood because the imputed $n_{it}^{\left(\text{𝓁}\right)}\;$is a positive, continuous rate, and showed no evidence of overdispersion. This model was fitted using the brms package (version 2.22.0). We ran four Markov chains per imputed dataset, each with 1000 warm-up iterations followed by 1000 sampling iterations, thinned to keep every 4 iterations, resulting in 1.6 million total posterior draws.

**Omicron onset timing.** The onset timing of Omicron emergence per province was defined as the first week in which the predicted cases exceeded a fixed case threshold of I being 30. For province i, the onset time was derived for each posterior draw ${\hat{\gamma }}_{i}^{\left(\text{𝓁}\right)}$ and ${\hat{\delta }}_{i}^{\left(\text{𝓁}\right)}$ as:


$$\begin{array}{c} \begin{array}{c} {\hat{T}}_{i}^{\left(\text{𝓁}\right)}=\frac{\text{log}\left(I\cdot \frac{\text{1}\text{0}\text{0},\text{0}\text{0}\text{0}}{p_{i}}\right)-{\hat{\gamma }}_{i}^{\left(\text{𝓁}\right)}}{{\hat{\delta }}_{i}^{\left(\text{𝓁}\right)}},\text{ 𝓁}=\text{1},\;\text{2},\;\ldots ,\;\text{1}.\text{6}\text{ million} \end{array} \end{array}$$ (9)

**Two association analyses (Step B3).** We did a Bayesian regression analysis to assess to which extent regional Omicron onset timing is associated with the geographical colleague connectivity patterns. From the full posterior of 1.6 million draws of onset times ${\hat{T}}_{i}^{\left(\text{𝓁}\right)}$, we randomly sampled 1000 draws and fit a Bayesian regression to each of the sample as indicated below.

For each province i, we summarized the connectivity derived in Section 2.1 to quantify the level of internal and external links. We decomposed links per residential province into two directional categories: (1) within-province links where both individuals live and work in province i, denoted as $d_{i}^{\text{wt}}$; (2) between-province links where at least one individual resides or works in another province, denoted as $d_{i}^{\text{bt}}$. Because multiplicative changes are more meaningful compared to additive changes in colleague connections, we worked with a logarithmic scale of the two types of connections and then standardized across provinces for better Hamiltonian Monte Carlo sampling efficiency,


$$\begin{array}{c} \tilde{d_{i}^{\text{wt}}}=\frac{{\text{log}}_{\text{2}}\left(d_{i}^{\text{wt}}\right)-\text{mean}\left({\text{log}}_{\text{2}}\left(d_{i}^{\text{wt}}\right)\right)}{\text{SD}\left({\text{log}}_{\text{2}}\left(d_{i}^{\text{wt}}\right)\right)} \end{array}$$

$$\begin{array}{c} \tilde{d_{i}^{\text{bt}}}=\frac{{\text{log}}_{\text{2}}\left(d_{i}^{\text{bt}}\right)-\text{mean}\left({\text{log}}_{\text{2}}\left(d_{i}^{\text{bt}}\right)\right)}{\text{SD}\left({\text{log}}_{\text{2}}\left(d_{i}^{\text{bt}}\right)\right)} \end{array}$$

We then fit the model for each draw of the estimated Omicron onset time for province i


$$\begin{array}{c} \begin{array}{c} {\hat{T}}_{i}^{\left(\text{𝓁}\right)}=\alpha_{\text{1}}+\tilde{\beta^{\text{wt}}}\cdot \tilde{d_{i}^{\text{wt}}}+\tilde{\beta^{\text{bt}}}\cdot \tilde{d_{i}^{\text{bt}}},\;\text{𝓁}=\text{1},\text{2},\ldots ,\;\text{1}\text{0}\text{0}\text{0} \end{array} \end{array}$$ (10)

where coefficients $\tilde{\beta^{\text{wt}}}$ and $\tilde{\beta^{\text{bt}}}$ represent the association with Omicron onset timing per one standard deviation increase in $\begin{array}{c} {\text{log}}_{\text{2}}\;\;\left(d_{i}^{\text{wt}}\right) \end{array}$ and ${\text{log}}_{\text{2}}\left(d_{i}^{\text{bt}}\right)$. To report the slopes on the unstandardized logarithmic scale, we transformed the coefficients as


$$\begin{array}{c} \beta^{\text{wt}}=\frac{\tilde{\beta^{\text{wt}}}}{SD\left({\text{log}}_{\text{2}}\left(d_{i}^{\text{wt}}\right)\right)} \end{array}$$

$$\begin{array}{c} \beta^{bt}=\frac{\tilde{\beta^{\text{bt}}}}{S\text{D}\left({\text{log}}_{\text{2}}\left(d_{i}^{\text{bt}}\right)\right)} \end{array}$$

Therefore, $\beta^{\text{wt}}$ and $\beta^{\text{bt}}\;$represent the change in Omicron onset timing associated with a two-fold increase in within-province and between-province colleague connections, respectively. Since onset timing was measured in weeks, we multiplied these transformed slopes by 7 to report the associations in days.

As Omicron was first seeded in the Netherlands through international flights from South Africa to the Netherlands, the onset of Omicron in Noord-Holland, where Amsterdam Airport Schiphol is located, was presumably driven by international transmissions instead of within-country mixing. Therefore, in addition to investigating the association between connectivity (with any province) and regional Omicron onset time, we also tested whether connectivity to Noord-Holland in particular, as a proxy for proximity to the primary importation hub, was associated with earlier local onset elsewhere. We computed for each residential province, number of colleague connections with individuals living in Noord-Holland, denoted as $d_{i}^{\text{NH}}$. Similarly, we first standardized the logarithmic of $d_{i}^{\text{NH}}$ for sampling efficiency:


$$\begin{array}{c} \tilde{d_{i}^{\text{NH}}}=\frac{{\text{log}}_{\text{2}}\left(d_{i}^{\text{NH}}\right)-\text{mean}\left({\text{log}}_{\text{2}}\left(d_{i}^{\text{NH}}\right)\right)}{\text{SD}\left({\text{log}}_{\text{2}}\left(d_{i}^{\text{NH}}\right)\right)} \end{array}$$

We fit the second model as:


$$\begin{array}{c} \begin{array}{c} {\hat{T}}_{i}^{\left(\text{𝓁}\right)}=\alpha_{\text{2}}+\tilde{\beta^{\text{NH}}}\cdot \tilde{d_{i}^{\text{NH}}},\text{𝓁}=\text{1},\text{2},\ldots ,\;\text{1}\text{0}\text{0}\text{0} \end{array} \end{array}$$ (11)

Again, we transform the coefficient to the unstandardized logarithmic scale as


$$\begin{array}{c} \beta^{\text{NH}}=\frac{\beta^{\tilde{N}H}}{\text{SD}\;\left({\text{log}}_{\text{2}}\;\left(d_{i}^{\text{NH}}\right)\right)} \end{array}$$

### 4.5. Implications for regional interventions (Step C)

Finally, we investigated how the geographical colleague connectivity patterns would change if two different regional interventions were in place. This potential impact of targeted interventions was quantified by the absolute count and proportion of colleague connections removed, both nationally and by residential province pair. If a region is in uniform lockdown, we removed all colleague links where at least one individual lived or worked in the targeted region. Alternatively, a cross-regional travel ban would only remove links involving individuals commuting into, or out of the targeted region, while keeping the links where both home and workplace were within that region. This travel-ban therefore preserves within-region workplace connectivity, which may allow essential onsite activities such as healthcare work and local supermarket supply to continue.

To assess how regional workforce size influences the potential intervention impact, we regressed the fraction of removed colleague links on regional workforce size. Regional workforce sizes were calculated by regional population aged over 15 years minus the unemployment counts, both accessed via CBS Open Data portal [40].

### 4.6. Sensitivity analyses

The cap D (i.e., the maximum number of colleagues an individual is allowed to have) was chosen from the values of 50, 100 and 200 for year 2019 and 2022. The threshold $e_{\text{min}}$, above which the start of the 4-week period to fit a loglinear increase in incident cases was defined, was chosen from 1, 3, 5 and 10. The case threshold I, above which the onset timing of Omicron emergence was defined, was chosen from 10, 20, 30, 100, 200 and 300. We repeated the branch assignment with 10 different random seeds and recomputed both the province-level composition of colleague connections and the full two-dimensional mixing matrix, comparing the results across the 10 seeds. For the province-level composition (shares that are within municipality, within province and between provinces, sum to 100%), we quantified sensitivity as the absolute difference, in percentage points, between the highest and lowest shares observed for each province across the 10 seeds. For the two-dimensional mixing matrix (raw connection counts), we quantified sensitivity as the relative difference between the highest and lowest value observed for each matrix entry across the 10 seeds, i.e., the difference between the maximum and minimum divided by the minimum.

## Supporting information

S1 Text Quantify geographical colleague connectivity by linking registry data files via CBS Microdata environment and assigning colleagues to local branches. (DOCX)

S1 Table A. Number of colleague links per residential province pair (logarithmic scale with 10 base, raw data used in Fig 2a). **B**. **Number of total colleague links per residential province (original and logarithmic scale with 10 base, raw data used in Fig 2b). C**. **Row-normalized colleague connections per residential province (raw data used in Fig 2c).**
(DOCX)

S2 Table Colleague connectivity shares by province and geographic scale (raw data used in Fig 3). (DOCX)

S3 Table Priors used in Bayesian analysis. (DOCX)

S4 Table Municipalities with the largest colleague link removal under regional interventions. (DOCX)

S1 Fig Probability of Omicron in sequenced SARS-CoV-2 cases per province. (DOCX)

S2 FigWeekly Omicron-positive cases per province ($e_{min}=1$).(DOCX)

S3 FigEstimated Omicron onset time per province (with the case threshold I=30).(DOCX)

S4 FigA. Sensitivity of Omicron onset time to the start of loglinear regression $e_{min}$.**B. Sensitivity of Omicron onset time ranking to the case threshold** I.(DOCX)

S5 Fig Association between connectivity with Noord-Holland and Omicron onset timing. (DOCX)

S6 Fig Reductions in colleague links per residential safety-region pair under safety-region-level lockdowns. (DOCX)

S7 Fig Comparison of the potential impact of two regional interventions on total national colleague connections. (DOCX)

S8 FigSensitivity of spatial colleague connectivity patterns to the degree cap D.(DOCX)
