# Supplementary material for: Mapping spatial colleague connectivity patterns from individual-level registry data to inform regional pandemic interventions
Source: PLoS Comput Biol. 2026 Aug 31;22(8):e1014721. doi: 10.1371/journal.pcbi.1014721 (PMC13568526; doi:10.1371/journal.pcbi.1014721)
Supplement: Supplementary file 1 [file pcbi.1014721.s001.docx]

**S1 Text. Quantify geographical colleague connectivity by linking registry data files via CBS Microdata environment and assigning colleagues to local branches.**

**Step 1**. Load registry data files from Statistics Netherlands (CBS) Microdata.

Three types of data files are loaded within CBS Microdata environment, they are: (1) job relationships (i.e., **SPOLISBUS**), (2) individuals’ home addresses (i.e., **GBAADRESOBJECT**, **VSLGTAB** and **GIN**), and (3) information on local branches (i.e., **GEMEENTESTPLTAB** and **ABR_REGIO**).

- **SPOLISBUS**: This is a database containing information on jobs and wages of employees at Dutch companies, available from the year 2010 onward. Each row in the database represents an income relationship, uniquely identified by the variable *IKVID*. Alongside *SBEID* (a unique identifier for an employer) and *RINPERSOON* (a unique identifier for an individual), each row captures the employment connection between an employer and an individual.
- **GBAADRESOBJECT**: This database contains the addresses of individuals registered with the municipality in the Personal Records Database. Each row links an individual (identified by the variable *RINPERSOON*) to a residential object (identified by a unique, meaningless number, *RINOBJECTNUMMER*). This residential object can be further linked to *VSLGTAB* to obtain the actual addresses. These data are available from year 1995 onwards upon request to CBS.
- **VSLGTAB**: This database contains the municipality code for inhabited objects, linked to their unique identifiers *RINOBJECTNUMMER*. Data are available from 1995 onwards.
- **GIN (GebiedeninNederland)**: This data file shows relationship between Dutch municipal divisions with higher-level administrative divisions of the Dutch territory. It merges municipal divisions with safety regions, which are administrative areas established to coordinate and manage emergency preparedness, response, and public safety measures.
- **GEMEENTESTPLTAB**: This database contains the municipality code associated with each job as of December in the reporting year, available from 2014 onward. Similar to SPOLISBUS, each row is uniquely identified by an income relationship (*IKVID*), along with identifiers for the person (*RINPERSOONS*) and their employer (*SBEID*). If multiple local branches belong to the same head company, only one *SBEID* is assigned. This database enhances the algorithm by providing additional information on an individual’s working municipality (*GEMEENTESTPL*). Consequently, the algorithm can establish connections among individuals working in the same local branch, rather than grouping them solely under the same head company.
- **ABR_REGIO**: This database provides additional, fine-grained information on companies. Specifically, it includes the number of local branches within the same municipality, recorded in the variable *Vest_Gemeente*.

**Step 2.** Merge loaded data files.

The loaded data files were linked using unique identifiers that are consistent across the databases. Through this linking process, information from multiple databases is combined and organized into a single data frame for further analysis.

- Step 2.1. **SPOLISBUS** was merged with **GBAADRESOBJECT** by linking the variable *RINPERSOON*, resulting in **SPO_GBA**. In this merged dataset, each individual in the colleague database is identified by a residential object number (i.e., *RINOBJECTNUMMER*).
- Step 2.2. **SPO_GBA** was merged further with **VSLGTAB** and **GIN** by linking *RINOBJECTNUMMER* and municipality code respectively, resulting in **SPO_ADR** where each individual in colleague database has information on home municipality and safety region.
- Step 2.3. To gather more detailed information on company structures, especially on which local branch each individual is (most probably) working in, **SPOLISBUS** was merged with **GEMEENTESTPLTAB** and **ABR_REGIO**. Company-level information is linked through mapping the unique job identification (i.e., *IKVID*). This merging process resulted in **SPO_ADR_LBE**, where each row represents a job relationship along with information on the individual’s home address (at both the municipality and safety region levels), working municipality, and the number of local branches belonging to the same head company within the same municipality.

**Step 3.** Pseudo code to compute colleague links per municipality triplet by assigning colleagues to local branches.

| **Input:** | | | | | |
| --- | --- | --- | --- | --- | --- |
|  | $(i, j, k):$ home municipalities $i, j$ and work municipality $k$ | | | | |
|  | $\left\{ C_{k} \right\}:$ set of head companies with at least one employee working in $k$ | | | | |
|  | $\left\{ B_{k}^{C} \right\}:$set of local branches of company $C_{k}$and operate in $k$ | | | | |
|  | $B_{k}^{C}=\left\vert\left\{ B_{k}^{C} \right\} \right\vert:$number of branches of company $C$and operate in $k$ | | | | |
|  | $N_{k}^{C}:$ total number of employees of $C$and work in $k$ (i.e., $N_{k}^{C}\geq1$) | | | | |
|  | $D:$maximum number of colleague links per individual is allowed to have (default $D=100$) | | | | |
| **Output:** | | | | | |
|  | $M_{k}^{i,j}:$ number of colleague pairs where colleagues live in municipalities $i$ and $j$, and work in $k$ | | | | |
| 1 | **for** a given municipality triplet $(i, j, k):$ | | | | |
| 2 |  | **for** each company $C_{k}\in\left\{ C_{k} \right\}$ | | | |
| 3 |  | | **if** $\left( N_{k}^{C}==1 \right)$ | | |
| 4 |  | |  | next | |
| 5 |  | | **end if** | | |
| 6 |  | | **if** (is.na$\left( B_{k}^{C} \right)$){ | | |
| 7 |  | |  | $B_{k}^{C}\leftarrow1\}$ | |
| 8 |  | | **end if** | | |
| 9 |  | | Assign each of the $N_{k}^{C}$ employees a branch label $b\in\left\{ 1,\ldots, B_{k}^{C} \right\}$ using random permutation | | |
| 10 |  | |  | of the vector formed by repeating $1,\ldots, B_{k}^{C}$ to length $N_{k}^{C}$ | |
| 11 |  | | **for** $\left( b=1,\ldots, B_{k}^{C} \right)\{$ | | |
| 12 |  | |  | $n_{b,k}\leftarrow$number of employees assigned to branch $b$ | |
| 13 |  | |  | Count employees living in municipality $i$ or $j$as $n_{b,k}^{i}$or $n_{b,k}^{j}$ | |
| 14 |  | |  | Compute colleague pairs by municipality triplet as: | |
| 15 |  | |  |  | $M_{b, k}^{i,j}=\left\{ \begin{aligned} n_{b,k}^{i}\cdot n_{b,k}^{j}, \text{if} i\neq j \\ \frac{n_{b,k}^{i}\cdot\left( n_{b,k}^{i}-1 \right)}{2}, \text{if} i=j \end{aligned} \right.$ |
| 16 |  | |  | Compute branch weight based on degree cap $D$: | |
| 17 |  | |  |  | $w_{b}=\left\{ \begin{aligned} \frac{D\cdot\left( D-1 \right)}{2M_{b, k}^{i,j}}, \text{if} n_{b,k}>D \\ 1, \text{if} n_{b,k}\leq D \end{aligned} \right.$ |
| 18 |  | | **end for** | | |
| 19 |  | **end for** | | | |
| 20 |  | Aggregate over all branches $b$ with its head company $C_{k}\in\left\{ C_{k} \right\}$ | | | |
| 21 |  |  | $\tilde{M}_{k}^{i,j}=\sum_{b} w_{b}\cdot M_{b,k}^{i,j}$ | | |
| 22 | **end for** | | | | |
