# Supplementary material for: Mapping spatial colleague connectivity patterns from individual-level registry data to inform regional pandemic interventions
Source: PLoS Comput Biol. 2026 Aug 31;22(8):e1014721. doi: 10.1371/journal.pcbi.1014721 (PMC13568526; doi:10.1371/journal.pcbi.1014721)
Supplement: Supplementary file 2 [file pcbi.1014721.s002.docx]

**S1 Table A. Number of colleague connections per residential province pair (logarithmic scale with 10 base, raw data used in Fig 2a).** This table presents the raw data used for generating Fig 2a, showing the number of colleague connections between residential province pairs. The data is transformed to a logarithmic scale (log base 10) to better visualize the heterogeneity between provinces. The order of the provinces in rows and columns are the same as how it is presented in Fig 2a. Full names of provinces and the corresponding abbreviations are shown in S1B Table.

|  | **GR** | **FR** | **DR** | **OV** | **FL** | **GD** | **UT** | **NH** | **ZH** | **ZL** | **NB** | **LB** |
| --- | --- | --- | --- | --- | --- | --- | --- | --- | --- | --- | --- | --- |
| **LB** | 4.39 | 4.46 | 4.44 | 4.88 | 4.48 | 5.65 | 5.11 | 5.26 | 5.38 | 4.45 | 6.20 | 6.84 |
| **NB** | 4.84 | 4.95 | 4.89 | 5.34 | 5.04 | 6.29 | 5.87 | 5.83 | 6.35 | 5.67 | 7.20 | 6.20 |
| **ZL** | 3.99 | 4.11 | 3.98 | 4.31 | 4.05 | 4.71 | 4.59 | 4.79 | 5.59 | 6.36 | 5.67 | 4.45 |
| **ZH** | 5.08 | 5.14 | 5.01 | 5.41 | 5.46 | 5.96 | 6.30 | 6.53 | 7.34 | 5.59 | 6.35 | 5.38 |
| **NH** | 5.19 | 5.40 | 5.03 | 5.46 | 6.18 | 5.91 | 6.40 | 7.20 | 6.53 | 4.79 | 5.83 | 5.26 |
| **UT** | 4.90 | 4.92 | 4.86 | 5.46 | 5.67 | 6.39 | 6.75 | 6.40 | 6.30 | 4.59 | 5.87 | 5.11 |
| **GD** | 5.04 | 5.13 | 5.25 | 6.32 | 5.68 | 7.06 | 6.39 | 5.91 | 5.96 | 4.71 | 6.29 | 5.65 |
| **FL** | 4.62 | 5.20 | 4.82 | 5.58 | 6.22 | 5.68 | 5.67 | 6.18 | 5.46 | 4.05 | 5.04 | 4.48 |
| **OV** | 5.21 | 5.43 | 5.89 | 6.85 | 5.58 | 6.32 | 5.46 | 5.46 | 5.41 | 4.31 | 5.34 | 4.88 |
| **DR** | 6.13 | 5.59 | 6.35 | 5.89 | 4.82 | 5.25 | 4.86 | 5.03 | 5.01 | 3.98 | 4.89 | 4.44 |
| **FR** | 5.81 | 6.60 | 5.59 | 5.43 | 5.20 | 5.13 | 4.92 | 5.40 | 5.14 | 4.11 | 4.95 | 4.46 |
| **GR** | 6.49 | 5.81 | 6.13 | 5.21 | 4.62 | 5.04 | 4.90 | 5.19 | 5.08 | 3.99 | 4.84 | 4.39 |

**S1 Table B. Number of total colleague links per residential province (original and logarithmic scale with 10 base, raw data used in Fig 2b).** This table presents the raw data used for generating Fig 2b, both the original and logarithmic scale with a base of 10.

| **Province** | **Number of all colleague connections** *(in million)* | **Logarithmic scale with base 10** |
| --- | --- | --- |
| **Groningen**  **(GR)** | 5.86 | 6.77 |
| **Fryslân**  **(FR)** | 6.14 | 6.79 |
| **Drenthe**  **(DR)** | 5.41 | 6.73 |
| **Overijssel**  **(OV)** | 11.88 | 7.07 |
| **Flevoland**  **(FL)** | 5.21 | 6.72 |
| **Gelderland**  **(GD)** | 21.03 | 7.32 |
| **Utrecht**  **(UT)** | 14.46 | 7.16 |
| **Noord-Holland**  **(NH)** | 25.90 | 7.41 |
| **Zuid-Holland**  **(ZH)** | 32.12 | 7.51 |
| **Zeeland**  **(ZL)** | 3.41 | 6.53 |
| **Noord-Brabant**  **(NB)** | 24.23 | 7.38 |
| **Limburg**  **(LB)** | 9.76 | 6.99 |

**S1 Table C. Row-normalized colleague links per residential province (raw data used in Fig 2c).** This table presents the raw data used for generating Fig 2c, showing the relative distribution of colleague connections from each residential province to another province. The data is transformed to a logarithmic scale with base 10 to better visualize the heterogeneity between provinces. The order of the provinces in rows and columns are the same as how it is presented in Fig 2c. Full names of provinces and the corresponding abbreviations are shown in S1 B Table

|  | **GR** | **FR** | **DR** | **OV** | **FL** | **GD** | **UT** | **NH** | **ZH** | **ZL** | **NB** | **LB** |
| --- | --- | --- | --- | --- | --- | --- | --- | --- | --- | --- | --- | --- |
| **LB** | 0.003 | 0.003 | 0.003 | 0.008 | 0.003 | 0.046 | 0.013 | 0.019 | 0.025 | 0.003 | 0.162 | 0.713 |
| **NB** | 0.003 | 0.004 | 0.003 | 0.009 | 0.005 | 0.081 | 0.031 | 0.028 | 0.092 | 0.019 | 0.661 | 0.065 |
| **ZL** | 0.003 | 0.004 | 0.003 | 0.006 | 0.003 | 0.015 | 0.011 | 0.018 | 0.115 | 0.676 | 0.138 | 0.008 |
| **ZH** | 0.004 | 0.004 | 0.003 | 0.008 | 0.009 | 0.028 | 0.062 | 0.105 | 0.688 | 0.012 | 0.069 | 0.007 |
| **NH** | 0.006 | 0.010 | 0.004 | 0.011 | 0.058 | 0.031 | 0.096 | 0.618 | 0.131 | 0.002 | 0.026 | 0.007 |
| **UT** | 0.005 | 0.006 | 0.005 | 0.020 | 0.032 | 0.171 | 0.388 | 0.172 | 0.137 | 0.003 | 0.052 | 0.009 |
| **GD** | 0.005 | 0.006 | 0.008 | 0.099 | 0.023 | 0.542 | 0.117 | 0.038 | 0.043 | 0.002 | 0.093 | 0.021 |
| **FL** | 0.008 | 0.031 | 0.013 | 0.072 | 0.321 | 0.093 | 0.090 | 0.288 | 0.056 | 0.002 | 0.021 | 0.006 |
| **OV** | 0.014 | 0.023 | 0.066 | 0.594 | 0.032 | 0.176 | 0.024 | 0.024 | 0.022 | 0.002 | 0.018 | 0.006 |
| **DR** | 0.248 | 0.072 | 0.419 | 0.144 | 0.012 | 0.033 | 0.013 | 0.020 | 0.019 | 0.002 | 0.014 | 0.005 |
| **FR** | 0.104 | 0.642 | 0.063 | 0.044 | 0.026 | 0.022 | 0.014 | 0.041 | 0.022 | 0.002 | 0.015 | 0.005 |
| **GR** | 0.531 | 0.109 | 0.228 | 0.028 | 0.007 | 0.019 | 0.013 | 0.026 | 0.020 | 0.002 | 0.012 | 0.004 |
