# Supplementary material for: Mapping spatial colleague connectivity patterns from individual-level registry data to inform regional pandemic interventions
Source: PLoS Comput Biol. 2026 Aug 31;22(8):e1014721. doi: 10.1371/journal.pcbi.1014721 (PMC13568526; doi:10.1371/journal.pcbi.1014721)
Supplement: Supplementary file 3 [file pcbi.1014721.s003.docx]

**S2 Table. Colleague link shares by province and geographic scale (raw data used in Fig 3).** This table presents the raw data used to plot Fig 3 in the paper, which shows the absolute numbers and proportions of colleague connections originating from individuals living within the same municipality (i.e. within municipality), different municipalities within the same province (i.e. within province), and different provinces (i.e. between provinces). Full names of provinces and the corresponding abbreviations are shown in S1.2 Table.

| **Province** | **N (all connections)**  *in million* | **N (within municipality)**  *in million* | **N (within province)**  *in million* | **N (between provinces)**  *in million* | **% (within municipality)** | **% (within province)** | **% (between provinces)** |
| --- | --- | --- | --- | --- | --- | --- | --- |
| **GR** | 5.86 | 1.51 | 1.60 | 2.75 | 25.7% | 27.4% | 46.9% |
| **FR** | 6.14 | 1.64 | 2.30 | 2.20 | 26.7% | 37.6% | 35.8% |
| **DR** | 5.41 | 1.16 | 1.10 | 3.14 | 21.5% | 20.4% | 58.2% |
| **OV** | 11.88 | 2.98 | 4.08 | 4.83 | 25.1% | 34.3% | 40.6% |
| **FL** | 5.21 | 1.23 | 0.45 | 3.54 | 23.6% | 8.6% | 67.9% |
| **GD** | 21.03 | 4.20 | 7.20 | 9.64 | 20.0% | 34.2% | 45.8% |
| **UT** | 14.46 | 2.35 | 3.27 | 8.84 | 16.2% | 22.6% | 61.2% |
| **NH** | 25.90 | 6.05 | 9.94 | 9.90 | 23.4% | 38.4% | 38.2% |
| **ZH** | 32.12 | 7.23 | 14.86 | 10.03 | 22.5% | 46.3% | 31.2% |
| **ZL** | 3.41 | 0.98 | 1.33 | 1.10 | 28.8% | 38.9% | 32.4% |
| **NB** | 24.23 | 5.35 | 10.65 | 8.22 | 22.1% | 44.0% | 33.9% |
| **LB** | 9.76 | 2.18 | 4.78 | 2.80 | 22.3% | 49.0% | 28.7% |
