# Supplementary material for: Mapping spatial colleague connectivity patterns from individual-level registry data to inform regional pandemic interventions
Source: PLoS Comput Biol. 2026 Aug 31;22(8):e1014721. doi: 10.1371/journal.pcbi.1014721 (PMC13568526; doi:10.1371/journal.pcbi.1014721)
Supplement: Supplementary file 4 [file pcbi.1014721.s004.docx]

**S3 Table. Priors used in Bayesian analysis.** Priors used for Bayesian inference framework to estimate provincial Omicron onset timing (i.e. logistic regression model and loglinear regression model), and the association analyses between Omicron onset and geographical colleague connectivity patterns.

| **Logistic regression model** | | |
| --- | --- | --- |
| $\mu_{\alpha}$ | Mean of provincial intercepts | Normal (1, 2) |
| $\sigma_{\alpha}$ | Standard deviation of provincial intercepts | Exponential (1) |
| $\mu_{\beta}$ | Mean of provincial slopes | Normal (5, 2) |
| $\sigma_{\beta}$ | Standard deviation of provincial slopes | Exponential (1) |
| **Loglinear regression model** | | |
| $\mu_{\gamma}$ | Mean of provincial intercepts | Normal (0, 0.1) |
| $\sigma_{\gamma}$ | Standard deviation of provincial intercepts | Exponential (1) |
| $\mu_{\delta}$ | Mean of provincial slopes | Normal (1, 0.5) |
| $\sigma_{\delta}$ | Standard deviation of provincial slopes | Exponential (1) |
| **Association analysis of within- and between-province connections** | | |
| $\alpha_{1}$ | Intercept (week number) | Normal (46, 2) |
| $\beta^{\text{wt}}$ | Effect of standardized log within-province connections | Normal (−0.5, 0.5) |
| $\beta^{bt}$ | Effect of standardized log between-province connections | Normal (−0.5, 0.5) |
| $\sigma_{1}$ | Standard deviation of the residuals | Exponential (2) |
| **Association analysis of connections with Noord-Holland (NH)** | | |
| $\alpha_{2}$ | Intercept (difference in weeks) | Normal (3, 0.5) |
| $\beta^{NH}$ | Effect of standardized log connections to Noord-Holland (NH) | Normal (−0.5, 0.5) |
| $\sigma_{2}$ | Standard deviation of the residuals | Exponential (2) |
