# Supplementary material for: Mapping spatial colleague connectivity patterns from individual-level registry data to inform regional pandemic interventions
Source: PLoS Comput Biol. 2026 Aug 31;22(8):e1014721. doi: 10.1371/journal.pcbi.1014721 (PMC13568526; doi:10.1371/journal.pcbi.1014721)
Supplement: Supplementary file 5 [file pcbi.1014721.s005.docx]

**S4 Table. Municipalities with the largest colleague link removal under regional interventions.** This table shows the ten municipalities with the highest number of colleague links removed under the two regional intervention scenarios, i.e. regional lockdown and cross-regional travel ban. The top ten cities are mostly the same under the two interventions, but the ranking differs. For example, Utrecht and Haarlemmermeer (in bold) both ranked higher in cross-regional travel ban than regional lockdown.

| **Intervention type** | **Municipality** | **N (removed links)**  *in million* | **% (total links removed)** |
| --- | --- | --- | --- |
| **Regional lockdown** | Amsterdam | 13.15 | 10.0 |
|  | Rotterdam | 10.00 | 7.6 |
|  | 's-Gravenhage | 6.85 | 5.2 |
|  | **Utrecht** | 6.60 | 5.0 |
|  | Eindhoven | 4.33 | 3.3 |
|  | Groningen | 3.64 | 2.8 |
|  | **Haarlemmermeer** | 3.54 | 2.7 |
|  | Tilburg | 3.47 | 2.6 |
|  | Almere | 3.18 | 2.4 |
|  | Breda | 3.09 | 2.3 |
| **Cross-regional travel ban** | Amsterdam | 10.87 | 8.2 |
|  | Rotterdam | 8.76 | 6.6 |
|  | **Utrecht** | 5.93 | 4.5 |
|  | 's-Gravenhage | 5.83 | 4.4 |
|  | Eindhoven | 3.83 | 2.9 |
|  | **Haarlemmermeer** | 3.31 | 2.5 |
|  | Groningen | 2.99 | 2.3 |
|  | Tilburg | 2.86 | 2.2 |
|  | Arnhem | 2.68 | 2.0 |
|  | Breda | 2.67 | 2.0 |
