# Supplementary material for: Mapping spatial colleague connectivity patterns from individual-level registry data to inform regional pandemic interventions
Source: PLoS Comput Biol. 2026 Aug 31;22(8):e1014721. doi: 10.1371/journal.pcbi.1014721 (PMC13568526; doi:10.1371/journal.pcbi.1014721)
Supplement: Supplementary file 6 [file pcbi.1014721.s006.docx]

**S1 Fig. Probability of Omicron in sequenced SARS-CoV-2 cases per province.** Observed proportions (green circles) represent the number of Omicron-positive sequences divided by the total number of sequenced SARS-CoV-2 cases per province. Posterior means and 95%CI from logistic regression model are shown as red lines and ribbons.
