# Supplementary material for: Mapping spatial colleague connectivity patterns from individual-level registry data to inform regional pandemic interventions
Source: PLoS Comput Biol. 2026 Aug 31;22(8):e1014721. doi: 10.1371/journal.pcbi.1014721 (PMC13568526; doi:10.1371/journal.pcbi.1014721)
Supplement: Supplementary file 7 [file pcbi.1014721.s007.docx]

**S2 Fig. Weekly Omicron-positive cases per province (**$\boldsymbol{e}_{\text{min}}\boldsymbol{=1}$**).** The estimated Omicron-positive incidence across provinces and time. The circles (red) and lines (blue) represent the posterior averages, the error bars (red) and ribbon areas (blue) show the 95% credible intervals for the logistic and loglinear model respectively. The start of the loglinear regression per province is defined as the first week when the posterior mean of expected Omicron new cases exceeded $e_{\text{min}}=1$. Lower bounds of the red bars were added by 0.1 if being 0, to allow meaningful logarithmic scale in the y axis.
