# Supplementary material for: Mapping spatial colleague connectivity patterns from individual-level registry data to inform regional pandemic interventions
Source: PLoS Comput Biol. 2026 Aug 31;22(8):e1014721. doi: 10.1371/journal.pcbi.1014721 (PMC13568526; doi:10.1371/journal.pcbi.1014721)
Supplement: Supplementary file 8 [file pcbi.1014721.s008.docx]

**S3 Fig. Estimated Omicron onset time per province (with the case threshold** $\boldsymbol{I=30}$**).** Posterior means (circles) and 95% credible intervals (error bars) indicate the estimated date in year 2021 when each province first reached 30 Omicron-positive cases.
