# Supplementary material for: Mapping spatial colleague connectivity patterns from individual-level registry data to inform regional pandemic interventions
Source: PLoS Comput Biol. 2026 Aug 31;22(8):e1014721. doi: 10.1371/journal.pcbi.1014721 (PMC13568526; doi:10.1371/journal.pcbi.1014721)
Supplement: Supplementary file 9 [file pcbi.1014721.s009.docx]

**S4 Fig A. Sensitivity of Omicron onset time to the start of loglinear regression** $\boldsymbol{e}_{\text{min}}$**.** Lines show the posterior mean ranking of each province’s Omicron onset across 4 different $e_{\text{min}}$values (based on 1.6 million posterior draws). On y axis, 1=earliest, 12=latest. Vertical bars show 95% credible intervals. The ranking pattern is stable across different values, with Noord-Holland consistently estimated as the first province experiencing Omicron onset.

**S4 Fig B. Sensitivity of Omicron onset time ranking to the case threshold** $\boldsymbol{I}$**.** Lines show the posterior mean ranking of each province’s Omicron onset across six thresholds for weekly Omicron-positive new cases. On y axis, 1=earliest, 12=latest. Vertical bars show 95% credible intervals. The ranking pattern is stable for thresholds equal or larger than 30 cases per week, with Noord-Holland consistently estimated as the first province experiencing Omicron onset. Only a few provinces changed rank when thresholds are 10 and 20, largely reflecting greater stochastic variability when case counts are low. The default choice of $I=30$ in the main analysis is a balance between the early-phase signalling and lower noise-driven mis-ranking.
