# Supplementary material for: Mapping spatial colleague connectivity patterns from individual-level registry data to inform regional pandemic interventions
Source: PLoS Comput Biol. 2026 Aug 31;22(8):e1014721. doi: 10.1371/journal.pcbi.1014721 (PMC13568526; doi:10.1371/journal.pcbi.1014721)
Supplement: Supplementary file 10 [file pcbi.1014721.s010.docx]

**S5 Fig. Association between connectivity with Noord-Holland and Omicron onset time.** A two-fold increase in colleague connections with residents of Noord-Holland was associated with an onset that was 2.0 days earlier (95% CI: 0.0 to 4.1 days). Full names of provinces and the corresponding abbreviations are shown in S1.2 Table.
