# Supplementary material for: Mapping spatial colleague connectivity patterns from individual-level registry data to inform regional pandemic interventions
Source: PLoS Comput Biol. 2026 Aug 31;22(8):e1014721. doi: 10.1371/journal.pcbi.1014721 (PMC13568526; doi:10.1371/journal.pcbi.1014721)
Supplement: Supplementary file 11 [file pcbi.1014721.s011.docx]

**S6 Fig. Reductions in colleague links per residential safety-region pair under safety-region-level lockdowns.** Each panel visualizes the relative reduction in colleague connection between residential safety-region pairs when each of the 25 safety regions labelled at the top of the panel is placed under lockdown. Panel order is consistent with safety regions from north to south. The darker the shades, the larger proportions of connections will be removed.
