# Supplementary material for: Mapping spatial colleague connectivity patterns from individual-level registry data to inform regional pandemic interventions
Source: PLoS Comput Biol. 2026 Aug 31;22(8):e1014721. doi: 10.1371/journal.pcbi.1014721 (PMC13568526; doi:10.1371/journal.pcbi.1014721)
Supplement: Supplementary file 12 [file pcbi.1014721.s012.docx]

**S7 Fig. Comparison of total national colleague link reductions under two regional interventions.** Fraction of total colleague links removed under a regional lockdown (x axis) compared to a cross-regional travel ban (y axis). Each circle indicates one of the 12 provinces (in blue), 25 safety regions (in orange) or 352 municipalities (in green). Grey dashed line indicates equal relative reductions under two regional interventions ($y=x)$. Compared to regional lockdowns whose relative reductions on total national colleague connections are largely driven by regional labour force size, high populated regions are less dominant under cross-regional travel bans that remove only cross-regional links. A few example provinces and municipalities are labelled.
