# Supplementary material for: Mapping spatial colleague connectivity patterns from individual-level registry data to inform regional pandemic interventions
Source: PLoS Comput Biol. 2026 Aug 31;22(8):e1014721. doi: 10.1371/journal.pcbi.1014721 (PMC13568526; doi:10.1371/journal.pcbi.1014721)
Supplement: Supplementary file 13 [file pcbi.1014721.s013.docx]

**S8 Fig. Sensitivity of spatial colleague connectivity patterns to the degree cap** $\boldsymbol{D}$**.** This figure shows the proportion of colleague links of the year 2022 that are within municipalities, within provinces, and between provinces under degree caps of 50, 100, and 200. As the degree cap increases, the proportion of within-municipality connections decreases by up to 5.24% (province ZL in the top panel), while the proportion of connections across provinces increases by up to 5.56% (province FL in the bottom panel). This pattern is expected, as using a higher degree cap allows for more connections in workplaces with many employees, which tend to connect individuals across wider geographical areas. Importantly, though the exact proportions per province across caps change, the contrast between provinces per cap remains similar.
