# Supplementary material for: Mapping spatial colleague connectivity patterns from individual-level registry data to inform regional pandemic interventions
Source: PLoS Comput Biol. 2026 Aug 31;22(8):e1014721. doi: 10.1371/journal.pcbi.1014721 (PMC13568526; doi:10.1371/journal.pcbi.1014721)
Supplement: Supplementary file 14 [file pcbi.1014721.s014.docx]

Overall, I think this is a strong and worthwhile manuscript, but its strongest contribution is methodological rather than epidemiological. For me the most valuable part of the paper is the construction of a fine-grained spatial workplace-connectivity dataset from national registry data. The municipality-triplet structure is useful and more informative than standard workplace contact surveys or simple commuting matrices.

In my view, the manuscript satisfies the PLOS Computational Biology criteria for originality, innovation, importance to the field, and methodological insight. The resulting data product could be valuable for spatial transmission models and for thinking more carefully about regionally targeted interventions.

Where I am less convinced is in the strength of the epidemiological interpretation. The Omicron analysis is interesting and plausible, but I see it as exploratory rather than strong evidence that colleague networks drove regional Omicron emergence. Similarly, the intervention analyses are useful, but they estimate reductions in inferred workplace links, not reductions in transmission or disease burden. I would therefore encourage the authors to frame the paper primarily as a methods/data-resource contribution with an illustrative epidemiological application.

The main issues I think should be addressed are:

- The paper should lean more explicitly into being a methods and data-resource paper. The Omicron analysis is useful as an illustration, but it should not be presented as if it validates workplace connectivity as a robust predictor of outbreak emergence.
- Temper causal or predictive language around Omicron. The association between colleague connectivity and earlier Omicron onset is plausible, especially for within-province connectivity, but the evidence is limited. The analysis is ecological, based on a small number of provinces, and vulnerable to confounding from international importation, work-from-home behaviour, schools/universities, household networks, testing intensity, and cross-border mobility.
- Treat Noord-Holland/Schiphol as a central complication, not a minor caveat. The paper itself notes that Noord-Holland likely experienced earlier Omicron onset because of international importation via Schiphol. That complicates the interpretation of domestic colleague connectivity as an explanation for early spread. The “connectivity to Noord-Holland” analysis is useful, but it should be framed more as proximity to an importation hub than as direct evidence of workplace-driven diffusion.
- Be clearer that colleague links are inferred opportunities for contact, not observed transmission-relevant contacts. A link between two colleagues does not tell us whether they met in person, how often they interacted, how long the contact lasted, whether they worked remotely, or what the workplace environment was like. This limitation is important enough that it should be emphasized earlier in the manuscript, not only in the limitations section.
- Give more justification for the degree cap of 100. The cap is reasonable as a way to avoid unrealistically connecting everyone in large workplaces, but the epidemiological meaning of 100 links per person is not obvious. The authors should clarify whether this represents a potential contact network over a certain time horizon, a pragmatic modelling convention, or a disclosure/computational choice. The sensitivity analyses should also be more visible in the main text.
- Address uncertainty around branch assignment. For companies with multiple branches, the manuscript has to infer which employees work at which local branch. If this assignment is wrong, the municipality-triplet matrix could be distorted. This may matter especially for large chains, hospitals, logistics firms, universities, and other multi-site employers. The authors should show whether headline spatial patterns are robust to alternative branch-assignment assumptions, or explain why major bias is unlikely.
- Interpret Figure 4 more critically. Figure 4 is interesting, but it also shows that connectivity does not explain all provincial onset timings well. The manuscript notes this, but I think it should go further by identifying which provinces are poorly explained and discussing what alternative mechanisms might account for them.
- Do not equate link removal with intervention effectiveness. The lockdown and travel-ban analyses estimate the proportion of inferred colleague links removed. They do not estimate infections prevented, reductions in $R$, hospitalizations avoided, or social/economic costs. The intervention section should consistently use language like “reduction in inferred workplace connectivity,” rather than implying direct epidemiological effectiveness.
- Make more of the strongest intervention results. The finding that different regions remove very different shares of national colleague links is one of the clearest and most convincing parts of the paper. The contrast between Zuid-Holland and Zeeland, and the finding that Amsterdam alone accounts for a large share of national colleague links, are concrete and policy-relevant. These results are stronger than the Omicron regression because they follow directly from the constructed network.
- Separate workers, links, and risk more carefully. A region with many workers is not necessarily the same as a region with many workplace links, and neither is necessarily the same as a region with high transmission risk. The Haarlemmermeer versus Almere example is useful because it shows that workplace location adds information beyond residential labour-force size. The paper should build more explicitly on this distinction.
- Acknowledge that missing sector and occupation information limits actionability. A workplace link in an office, hospital, warehouse, school, airport, restaurant, or factory does not carry the same transmission risk. Because the paper is interested in targeted interventions, the lack of sector, occupation, contract type, and remote-work potential is a meaningful limitation.
- Improve usability of the figures/results. Some of the figures, especially the chord plots, are informative but hard to translate into decisions. I would recommend adding ranked tables of the largest home-work flows, largest home-home colleague connections, municipalities with the largest lockdown effect, and municipalities with the largest cross-regional travel-ban effect.
- Tone down the conclusion. The manuscript can strongly conclude that registry-derived workplace connectivity is a valuable spatial input for future transmission modelling and policy evaluation. It should be more cautious about implying that the current analysis can identify where outbreaks will occur earlier or which interventions will be most epidemiologically effective.

Overall, I would support publication after revision. The manuscript provides substantial evidence for its conclusions about spatial workplace connectivity and the uneven effects of regional interventions on inferred colleague links. The evidence for conclusions about Omicron onset and outbreak risk is more suggestive, and the paper would be stronger if that distinction were made more explicit throughout.
