# Supplementary material for: Mapping spatial colleague connectivity patterns from individual-level registry data to inform regional pandemic interventions
Source: PLoS Comput Biol. 2026 Aug 31;22(8):e1014721. doi: 10.1371/journal.pcbi.1014721 (PMC13568526; doi:10.1371/journal.pcbi.1014721)
Supplement: Supplementary file 15 [file pcbi.1014721.s016.docx]

# Response to reviewer comments

We thank the reviewers for their constructive comments and feedback. Overall, the reviewers agree on the importance and relevance of this study's findings, but also suggest several rewordings and additional analyses. We have therefore added additional sensitivity analyses, revised the manuscript to clarify the interpretation of the Omicron association analysis and intervention scenarios, and toned down statements that could imply causality, prediction, or direct epidemiological effectiveness.

Below, we address each reviewer comment in detail, where we list our responses in grey boxes, like here. In the package of resubmitted manuscript files, we also include a copy of the manuscript with tracked changes.

## Reviewer #1

Overall, I think this is a strong and worthwhile manuscript, but its strongest contribution is methodological rather than epidemiological. For me the most valuable part of the paper is the construction of a fine-grained spatial workplace-connectivity dataset from national registry data. The municipality-triplet structure is useful and more informative than standard workplace contact surveys or simple commuting matrices.

In my view, the manuscript satisfies the PLOS Computational Biology criteria for originality, innovation, importance to the field, and methodological insight. The resulting data product could be valuable for spatial transmission models and for thinking more carefully about regionally targeted interventions.

Where I am less convinced is in the strength of the epidemiological interpretation. The Omicron analysis is interesting and plausible, but I see it as exploratory rather than strong evidence that colleague networks drove regional Omicron emergence. Similarly, the intervention analyses are useful, but they estimate reductions in inferred workplace links, not reductions in transmission or disease burden. I would therefore encourage the authors to frame the paper primarily as a methods/data-resource contribution with an illustrative epidemiological application.

The main issues I think should be addressed are:

1. The paper should lean more explicitly into being a methods and data-resource paper. The Omicron analysis is useful as an illustration, but it should not be presented as if it validates workplace connectivity as a robust predictor of outbreak emergence.

Response 1

Thank you for this suggestion. We agree that the main contributions of this paper are the reuseable data product and the generalizable workflow to derive the spatial connectedness from individual-level registry data. As such, we have revised part of the *Abstract, Introduction, Discussion and Methods* (regarding Omicron association analyses) to lean more explicitly on being a methods and data-resource paper and to avoid suggesting too much the potential epidemiological impacts. One further update related to the reporting of the association analyses: rather than transforming the connection counts to a natural-logarithmic scale and standardizing them (equations 9–11), we now applied a base-2 logarithmic transformation and reported the estimated slope parameters on the non-standardized base-2 logarithmic scale, as also described in response 19 (Reviewer 2). This has not changed the substantial contents of the results or the conclusions. The following revised texts have additions in bold and removed parts with strikethrough:

*ABSTRACT: We* ***illustrate*** *~~demonstrate~~ the* ***epidemiological relevance*** *~~utility~~ of this spatial connectivity ~~in signalling regions with elevated outbreak risks. Using~~* ***by using*** *SARS-CoV-2 Omicron as an example: a ~~ten-fold~~* ***two-fold*** *increase in within-province connections was associated with a ~~12~~****3.7****-day earlier (95% CI: ~~2~~* ***0.6*** *to ~~22~~* ***6.6*** *days) Omicron onset, and between-province connectivity was associated with an ~~8~~* ***2.5****-day earlier (95% CI: ~~-4~~* ***-1.0*** *to ~~21~~* ***6.2*** *days) onset. ~~These results suggest that the impact of regional interventions shifting spatial connectivity patterns should be expected to vary by region and type of intervention.~~* ***We also quantified the number of colleague connections that would be removed in case of regional mobility restrictions such as a lockdown.*** *~~Together,~~ ~~our findings draw attention of using this~~* ***In future modelling studies, these*** *highly fine-grained spatial connectivity* ***data*** ***could be used as spatial mixing matrices to more explicitly capture the connectedness and dependency between regions to inform more*** *~~to enable more regionally~~ tailored ~~and network-targeted~~ policy measures.*

*INTRODUCTION: Building on these connectivity patterns, we ~~assessed their epidemiological relevance by applying Bayesian inference to demonstrate associations between colleague connectedness and provincial onset timing of SARS-CoV-2 Omicron emergence~~* ***further estimated regional SARS-CoV-2 Omicron onset timing and investigated whether the colleague connectivity was associated with regional onset of Omicron emergence****.*

*DISCUSSION: ~~We show that this connectedness can flag regions with elevated risks of a subsequent infection wave, by demonstrating associations between connectivity patterns and SARS-CoV-2 Omicron onset timing.~~* ***It is reassuring that our hypothesis of colleague connectivity being relevant to spatial outbreak dynamics is supported by the association between connectivity patterns and regional SARS-CoV-2 Omicron onset timing.*** *We further ~~showcased the utility of the derived data product in supporting regionally tailored interventions, using a regional lockdown and a cross-regional travel ban as examples~~* ***quantified reductions in colleague connections under two regional interventions. These reductions can serve as inputs for adjusting spatial mixing matrices in future transmission models which aim to quantify the epidemiological impact of regionally tailored interventions.***

*METHODS: We did a Bayesian regression analysis to ~~validate~~* ***assess*** *to which extent regional Omicron onset timing is associated with the geographical colleague connectivity patterns.*

2. Temper causal or predictive language around Omicron. The association between colleague connectivity and earlier Omicron onset is plausible, especially for within-province connectivity, but the evidence is limited. The analysis is ecological, based on a small number of provinces, and vulnerable to confounding from international importation, work-from-home behaviour, schools/universities, household networks, testing intensity, and cross-border mobility.

Response 2

We understand the reviewer's concern. We did not intend to frame the association analyses as causal or predictive, and for this exact reason already carefully used the term "association" throughout instead of language implying causal relationships such as "relation" or "connectivity drives the spread of Omicron".

Still, we carefully checked the full manuscript to avoid language implying a stronger claim than intended. These revisions are partly described in Response 1. For example, we replaced wording like "to validate", "to flag regions with elevated risks of a subsequent infection wave" with "to assess" and "to check the plausible role of connectivity in regional outbreak dynamics", and we avoided implying that the associations are predictive enough to "support" the design of regional interventions. In addition, we made the following revision:

*DISCUSSION: Geographical colleague connectivity patterns ~~explain~~* ***are correlated with*** *part of the variation in provincial Omicron onset, ~~but not all~~* ***though other factors are likely to contribute as well.***

3. Treat Noord-Holland/Schiphol as a central complication, not a minor caveat. The paper itself notes that Noord-Holland likely experienced earlier Omicron onset because of international importation via Schiphol. That complicates the interpretation of domestic colleague connectivity as an explanation for early spread. The “connectivity to Noord-Holland” analysis is useful, but it should be framed more as proximity to an importation hub than as direct evidence of workplace-driven diffusion.

Response 3

We agree that such a clear distinction of the complication of Noord-Holland is correct and necessary. We therefore reframed the "connectivity to Noord-Holland" analysis as proximity to an importation hub, instead of "workplace connections to Noord-Holland" where possible. For example:

*METHODS: As Omicron was first seeded in the Netherlands through international flights from South Africa to the Netherlands, the onset of Omicron in Noord-Holland, where Amsterdam Airport Schiphol is located, was* ***presumably*** *driven by international transmissions instead of within-country mixing.* ***Therefore, in addition to investigating the association between connectivity (with any province) and regional Omicron onset time, we also*** *tested whether* ***connectivity*** *to Noord-Holland* ***in particular, as a proxy for proximity to the primary importation hub,*** *was associated with earlier local onset.*

*RESULTS: To ~~probe~~* ***investigate*** *the association with case importation from international travel hubs such as Amsterdam Airport Schiphol in Noord-Holland, we show ~~in supplementary Fig S5~~ that* ***colleague connectivity to Noord-Holland, interpreted here as a proxy for proximity to the importation hub, is negatively associated with regional Omicron onset timing (supplementary Fig S5):*** *a* ***two****-fold increase in connections with Noord-Holland was associated with an onset that was* ***2.0 days earlier (95% CI: 0.0 to 4.1 days)*** *~~a 7-day earlier on the onset timing of local outbreaks (95% CI: 0 to 14 days earlier), as Noord-Holland (NH) is estimated to experience the earliest Omicron likely due to international flights arriving at Amsterdam Airport Schiphol~~.*

4. Be clearer that colleague links are inferred opportunities for contact, not observed transmission-relevant contacts. A link between two colleagues does not tell us whether they met in person, how often they interacted, how long the contact lasted, whether they worked remotely, or what the workplace environment was like. This limitation is important enough that it should be emphasized earlier in the manuscript, not only in the limitations section.

Response 4

We revised a sentence in the first paragraph of the Results section to clearly distinguish workplace links with transmission-relevant contacts (additions in bold). We also added an explicit definition of the inferred colleague links in the Methods section. We then checked the manuscript throughout to avoid implying that inferred colleague links represent direct contacts or meetings in the workplaces.

*RESULTS*: *The number of individuals working in the Netherlands with valid payroll tax registrations ranged from 8.0 million in year 2019 to 8.5 million in 2022. We paired colleagues working for the same company branch and counted undirected colleague links per municipality triplet. The colleague link counts****, representing cumulative opportunities for workplace contact,*** *were denoted as* $M_{ij}^{k}$*, with* $i$ *and* $j$ *being the two colleagues’ residential municipalities and* $k$ *the municipality of their shared workplace.*

*METHODS:* *Then we linked individuals’ residential municipalities to the municipality where they work, to quantify the number of work-related connections per municipality triplet (two home municipalities for two individuals and their shared working municipality).* ***These links identified by shared workplace represented cumulative opportunities for contact, rather than transmission-relevant contacts capturing the frequency or duration of in-person interactions.***

*RESULTS:* ***For the regional lockdown scenario, each panel in*** *Fig 5 shows the ~~effect on the spatial colleague connectivity~~* ***relative reductions of colleague connections*** *per residential province pair ~~(same structure as in Fig 2a)~~ ~~if~~* ***when*** *different provinces are in lockdown. ~~Each panel places one province under lockdown and maps the relative reduction of colleague connections per residential province pair.~~* ***The panels*** *~~Panels~~ are symmetric because ~~colleague links are undirected, regardless of which residential province is listed first~~* ***removed links between residents of province A and province B were counted the same as links between residents of province B and province A, the same structure of Fig 2a.*** *~~As expected,~~ Colleague connections ~~among residents~~* ***of people living in*** *~~of~~ the locked province ~~are~~* ***were*** *entirely removed ~~regardless of the meeting province~~, clearly shown by the darkest shades in all panels. Off-diagonal shading reflects* ***the reduction*** *in connections between* ***colleagues that both live in a province that is not locked down and are linked through a workplace in*** *the locked province.*

5. Give more justification for the degree cap of 100. The cap is reasonable as a way to avoid unrealistically connecting everyone in large workplaces, but the epidemiological meaning of 100 links per person is not obvious. The authors should clarify whether this represents a potential contact network over a certain time horizon, a pragmatic modelling convention, or a disclosure/computational choice. The sensitivity analyses should also be more visible in the main text.

Response 5

Regarding a clarification of the choice of the degree cap being 100, we modified texts in the Method section (where the cap was first defined) to clarify that the cap represents the maximum number of epidemiologically relevant workplace links over the employment period:

*METHODS: We therefore let each employee have a degree cap* $D$*as the maximum number of colleague connections per individual is allowed to have* ***over the employment period****. We followed the decision of Statistics Netherlands (CBS) and set as default:* $D=100$*(1).* ***This order of magnitude aligns with Dunbar's theory of a cognitive limit on human group size being 148 as having stable relationships (2).***

Regarding the sensitivity analysis of the cap, we now describe an additional analysis using three degree caps: 50, 100, and 200, based on the 2022 data. This sensitivity analysis is now included as a supplementary figure, with corresponding text added to the Results section:

*RESULTS 2.4. Sensitivity analysis: The spatial connectivity patterns ~~are stable~~* ***show broadly consistent province-level contrasts*** *across different choices of the degree cap*$D$*(i.e., the maximum number of colleague connections an individual is allowed to have****)****,* ***although the total number of inferred colleague links (ranged from 61 million to 239 million) and their spatial compositions changed with the choice of cap (supplementary Fig S8)*** *~~datasets presented online (3))~~.* ***As the cap increased from 50 to 200, the percentage of between-province connections rose by up to 5.56%, at the expense of within-municipality links.***

Figure and captions added to the *Supplementary Information*:

*"Fig S8: Sensitivity of spatial colleague connectivity patterns to the degree cap* $D$*.* *The figure shows the proportion of colleague links of the year 2022 that are within municipalities, within provinces, and between provinces under degree caps of 50, 100, and 200. As the degree cap increases, the proportion of within-municipality connections decreases by up to 5.24% (province ZL in the top panel), while the proportion of connections across provinces increases by up to 5.56% (province FL in the bottom panel). This pattern is expected, as using a higher degree cap allows for more connections in workplaces with many employees, which tend to connect individuals across wider geographical areas. Importantly, though the exact proportions per province across caps change, the contrast between provinces per cap remains similar."*

6. Address uncertainty around branch assignment. For companies with multiple branches, the manuscript has to infer which employees work at which local branch. If this assignment is wrong, the municipality-triplet matrix could be distorted. This may matter especially for large chains, hospitals, logistics firms, universities, and other multi-site employers. The authors should show whether headline spatial patterns are robust to alternative branch-assignment assumptions, or explain why major bias is unlikely.

Response 6

Based on this suggestion, we now addressed the uncertainty of the headline spatial patterns around the two key assumptions regarding the branch-assignment algorithm, namely the random assignment and the near-even split. For each assumption, we added the results and methodology to the *Results* and the *Methods* sections, respectively.

Regarding the random assignment:

For companies with multiple local branches within the same municipality, no information was available on which employee worked at which local branch. As described in the *Methods* section (Section 4.2. Construction of geographical colleague connectivity patterns), we assigned each employee to one of the local branches at random using a random seed. We do not have information to support an alternative assumption other than random assignment. To address the sensitivity around the arbitrary choice of the random seed, we now repeated the branch assignment with 10 different random seeds and recomputed the spatial connectivity patterns. We added the following text to the *Results* section, after the grey texts describing the sensitivity to different choices of the cap as described in Response 5:

*RESULTS 2.4. Sensitivity analysis: The spatial connectivity patterns ~~are stable~~* ***show broadly consistent province-level contrasts*** *across different choices of the degree cap*$D$*(i.e., the maximum number of colleague connections an individual is allowed to have****)****,* ***although the total number of inferred colleague links (ranged from 61 million to 239 million) and their spatial compositions changed with the choice of cap (supplementary Fig S8)*** *~~datasets presented online (3))~~.* ***As the cap increased from 50 to 200, the percentage of between-province connections rose by up to 5.56%, at the expense of within-municipality links.*** ***These patterns were also robust to the arbitrary choice of random seed used to assign employees to local branches. Across 10 different random seeds, the province-level shares of within-municipality, within-province, and between-province colleague connections (same metric as presented in Fig 3) varied by at most 0.10 percentage points. And the largest relative difference across the 10 seeds for any single entry of the two-dimensional mixing matrix (same metric as presented in Fig 2a) was 1.99%.***

And we added the following texts to the *Methods* section:

*METHODS 4.5. Sensitivity analysis:* ***We repeated the branch assignment with 10 different random seeds and recomputed both the province-level composition of colleague connections and the full two-dimensional mixing matrix, comparing the results across the 10 seeds. For the province-level composition (shares that are within municipality, within province and between provinces, sum to 100%), we quantified sensitivity as the absolute difference, in percentage points, between the highest and lowest shares observed for each province across the 10 seeds. For the two-dimensional mixing matrix (raw connection counts), we quantified sensitivity as the relative difference between the highest and lowest value observed for each matrix entry across the 10 seeds, i.e. the difference between the maximum and minimum divided by the minimum.***

Regarding the near-even split:

For companies with multiple local branches within the same municipality, we also assumed that different branches had approximately equal numbers of employees. We have now added a discussion of this assumption as one of the limitations in the *Discussion* section:

*DISCUSSION: Our study has ~~four~~* ***five*** *limitations, for which we describe the consequences and planned mitigation steps below. …* ***Third, under the near-even split assumption, we assumed that different branches of the same company within the same municipality have approximately equal numbers of employees. In the absence of the degree cap, assigning the same total number of employees to the same number of local branches evenly resulted in an underestimated total colleague links compared to the more skewed branch size distribution. However, with a cap on the maximum number of connections per person, this bias was reversed for companies where the average number of employees per branch was around this cap value.***

7. Interpret Figure 4 more critically. Figure 4 is interesting, but it also shows that connectivity does not explain all provincial onset timings well. The manuscript notes this, but I think it should go further by identifying which provinces are poorly explained and discussing what alternative mechanisms might account for them.

Response 7

We agree that Fig 4 should be thoroughly and critically discussed, especially the provinces that deviated markedly from the overall pattern. Due to this specific reason, we have explicitly named a few examples and discussed alternative mechanisms in the Discussion section (starting with "*For example, even though Flevoland (FL) …".*). To note this key … earlier in the manuscript, we now added the two examples in the Results section while keeping the extensive discussion of alternative mechanisms in the Discussion section.

*RESULTS:* *In both panels of Fig 4,* ***some provinces deviate markedly from the overall pattern*** *~~not all provinces’ Omicron onset timing fall within the uncertainty region of the fitted regression, suggesting that onset timing is only partially explained by within- or between-province colleague connections~~.* ***For example, Overijssel (OV) had later Omicron emergence than the pattern would suggest. In contrast, Gelderland (GD) experienced an earlier Omicron onset than expected from both the within-province and between-province connectivity regressions.***

*DISCUSSION: Geographical colleague connectivity patterns ~~explain~~* ***are correlated with*** *part of the variation in provincial Omicron onset, but not all* ***though other factors are likely to contribute as well.*** For example, even though Flevoland (FL) *~~is~~* ***was*** highly connected to Noord-Holland (NH), ~~its~~ Omicron ***emerged*** ~~onset was~~ much later than ~~the latter~~ **the overall pattern (Fig S5)**. This might be explained by the **first** association analysis**,** where Flevoland (FL) has relatively fewer within-province colleague connections ***(Fig 4a)***. ~~Another reason might be that, at the time of the Omicron outbreak, many companies in Noord-Holland (NH) had a working-from-home policy reducing the level of onsite mixing.~~ ***Overijssel also had a late Omicron emergence, but instead of lacking within- or between-province colleague links, an alternative mechanism might be that our association models do not capture whether each region is linked to the region of higher or lower transmission risks.*** In contrast, Gelderland (GD) ~~shows~~ **experienced** a relatively early estimated Omicron onset~~, but it does not have that many colleague connections within-province, between-province, or to Noord-Holland (NH)~~. A plausible explanation might be the substantial ~~classmate~~ ~~connections~~ ***student mixing*** ~~around~~ **surrounding** its university hub**s *such as Wageningen University and Radboud University.***

8. Do not equate link removal with intervention effectiveness. The lockdown and travel-ban analyses estimate the proportion of inferred colleague links removed. They do not estimate infections prevented, reductions in $R$, hospitalizations avoided, or social/economic costs. The intervention section should consistently use language like “reduction in inferred workplace connectivity,” rather than implying direct epidemiological effectiveness.

Response 8

Thank you for highlighting this important distinction. We have revised the manuscript throughout to use consistent language such as “reduction in workplace links" to avoid implications of epidemiological impacts. For example, the titles of Fig 5 to 7, S6 to S7, and the following Results section:

*RESULTS:* *After aggregating the removed connections to the national level****,*** *~~(i.e.~~ the fraction~~s~~ of total colleague connections removed under a regional lockdown~~), the potential impacts of regional lockdowns~~ was unevenly distributed across regions (Fig 6).* … *~~For~~* ***When locking down*** *municipalities,* ***the proportion of removed workplace links*** *~~potential impact~~ is highly skewed (green bars).*

9. Make more of the strongest intervention results. The finding that different regions remove very different shares of national colleague links is one of the clearest and most convincing parts of the paper. The contrast between Zuid-Holland and Zeeland, and the finding that Amsterdam alone accounts for a large share of national colleague links, are concrete and policy-relevant. These results are stronger than the Omicron regression because they follow directly from the constructed network.

Response 9

Thank you for raising this. We have added in the last part of the abstract to emphasize more the variations in removed colleague connections under regional intervention scenarios.

**Additions in the *Abstract* section:**

*"Based on our estimates of spatial connectivity, we quantified the number of colleague connections that would be removed in case of regional mobility restrictions such as a lockdown: locking down the whole province Zeeland would remove 2.6% of colleague links at the national level while the city Amsterdam alone would remove 10.0%."*

10. Separate workers, links, and risk more carefully. A region with many workers is not necessarily the same as a region with many workplace links, and neither is necessarily the same as a region with high transmission risk. The Haarlemmermeer versus Almere example is useful because it shows that workplace location adds information beyond residential labour-force size. The paper should build more explicitly on this distinction.

Response 10

We understand the reviewer's concern and agree that an explicit distinction among workforce size, inferred workplace links and transmission risks is necessary. Therefore, we checked and revised the manuscript where these concepts were blended or misapplied. For example:

*DISCUSSION*: We ~~are aware~~ **noted** that ~~part of the heterogeneous impact of~~ **the fraction of colleague links removed under** a regional lockdown ~~overlaps~~ **is related to** ~~with~~ the labour force size per administrative unit, as regions with more workers ~~would have proportionally~~ **generally** **contribute more** ~~working-age individuals that contribute largely~~ to the national total ~~colleague~~ **workplace** link**s** ~~counts~~. **However, workforce size alone does not determine the volume of workplace links, but also factors such as the number and size distribution of workplaces. Moreover, the number of workplace links does not shape transmission risks alone; the latter also depends on transmission-related characteristics such as in-person contact intensity and remote-work patterns.**

11. Acknowledge that missing sector and occupation information limits actionability. A workplace link in an office, hospital, warehouse, school, airport, restaurant, or factory does not carry the same transmission risk. Because the paper is interested in targeted interventions, the lack of sector, occupation, contract type, and remote-work potential is a meaningful limitation.

Response 11

Regarding missing sector, occupation and further granularity, we have expanded the following limitation to make this point more explicit.

*DISCUSSION*: *Third, ~~current colleague connectivity has not explicitly included granularity on~~* ***our results currently do not consider specific*** *industrial sectors****,*** ***occupation,*** *~~or by~~ types of contracts****, or remote-work potential*** *~~therefore limiting the design of possible intervention strategies (such as targeting on certain sectors)~~.* **To explore the effects of interventions targeting specific sectors or occupations, our analyses would need to be further stratified.**

12. Improve usability of the figures/results. Some of the figures, especially the chord plots, are informative but hard to translate into decisions. I would recommend adding ranked tables of the largest home-work flows, largest home-home colleague connections, municipalities with the largest lockdown effect, and municipalities with the largest cross-regional travel-ban effect.

Response 12

We agree that improving the usability and interpretability of the figures and results is important. We have therefore made two changes: (1) we added explanatory text to guide the interpretation of the chord plots, and (2) we added a supplementary ranking table for the municipalities most affected by regional mobility-restricted interventions.

(1) Chord plots. We carefully considered the reviewer's suggestion to add ranked tables of the largest home-work flows and home-home connections but decided to retain the chord plots (Fig 1) as the primary presentation. Our reason is that the top ranking of region pairs with largest workplace links is dominated by within-province connections for both home-work flows and home-home connections. In contrast, the chord plots show both the dominance of within-province connections and the non-negligible, heterogeneous between-province patterns, which would be less visible in a ranked table. To interpret the chord plots more effectively: we added a leading sentence in the Result section where Fig 1 was introduced.

**Additions in the *Result* section:**

*"Next, we show the extent to which geographical colleague connectivity varies across regions, both in terms of where residents work and where their workplace colleagues live."*

(2) Ranked tables of the municipalities with the largest lockdown and cross-regional travel-ban effects. We agree with the reviewer that indeed, a ranked table would help identify regions with a larger role in total colleague links. Therefore, we summarized the municipalities with the largest workplace link removal under the regional-lockdown and cross-regional travel-ban scenarios. This ranked table is now included as a supplementary table (Table S4), with texts added to the Results section to refer to this table.

**Additions in the *Result* section:**

*"The municipalities with the largest colleague-link removal were mainly large residential and employment centers, with rankings differing between the two intervention types (Supplementary Table S4)."*

**Additions in the S*upplementary Information*:**

*"Table S4. Municipalities with the largest colleague link removal under regional interventions.* *This table shows the ten municipalities with the highest number of colleague links removed under the two regional intervention scenarios, i.e. regional lockdown and cross-regional travel ban. The top ten cities are mostly the same under the two interventions, but the ranking differs. For example, Utrecht and Haarlemmermeer (in bold) both ranked higher in cross-regional travel ban than regional lockdown."*

| **Intervention type** | **Municipality** | **N (removed links)**  *in million* | **% (total links removed)** |
| --- | --- | --- | --- |
| **Regional lockdown** | Amsterdam | 13.15 | 10.0 |
|  | Rotterdam | 10.00 | 7.6 |
|  | 's-Gravenhage | 6.85 | 5.2 |
|  | **Utrecht** | 6.60 | 5.0 |
|  | Eindhoven | 4.33 | 3.3 |
|  | Groningen | 3.64 | 2.8 |
|  | **Haarlemmermeer** | 3.54 | 2.7 |
|  | Tilburg | 3.47 | 2.6 |
|  | Almere | 3.18 | 2.4 |
|  | Breda | 3.09 | 2.3 |
| **Cross-regional travel ban** | Amsterdam | 10.87 | 8.2 |
|  | Rotterdam | 8.76 | 6.6 |
|  | **Utrecht** | 5.93 | 4.5 |
|  | 's-Gravenhage | 5.83 | 4.4 |
|  | Eindhoven | 3.83 | 2.9 |
|  | **Haarlemmermeer** | 3.31 | 2.5 |
|  | Groningen | 2.99 | 2.3 |
|  | Tilburg | 2.86 | 2.2 |
|  | Arnhem | 2.68 | 2.0 |
|  | Breda | 2.67 | 2.0 |

13. Tone down the conclusion. The manuscript can strongly conclude that registry-derived workplace connectivity is a valuable spatial input for future transmission modelling and policy evaluation. It should be more cautious about implying that the current analysis can identify where outbreaks will occur earlier or which interventions will be most epidemiologically effective.

Response 13

We thank the reviewer again for pointing out the necessity to reword the Omicron association analysis to avoid implications of causality or prediction. We have addressed these issues in our responses to Comments 1 and 2. Here we further toned down the conclusion by clarifying that the Omicron analysis provides evidence of association, while future modelling is necessary to quantify the epidemiological impacts (additions in bold, removed texts with strikethrough).

*DISCUSSION:* *In conclusion, we establish a reusable data product of geographical connectedness in colleague networks****. This workplace connectivity provides a valuable spatial input for future transmission modelling and policy evaluation, particularly for representing how workplace links are distributed within and between regions.*** *~~and confirm its utility in designing and evaluating more regionally targeted and social-network tailored pandemic measures.~~* ***The Omicron analysis reassures that how such connectivity patterns are associated with regional differences in outbreak timing; further dynamic transmission modelling is needed to assess the impact of interventions that affect colleague connectivity.***

14. Overall, I would support publication after revision. The manuscript provides substantial evidence for its conclusions about spatial workplace connectivity and the uneven effects of regional interventions on inferred colleague links. The evidence for conclusions about Omicron onset and outbreak risk is more suggestive, and the paper would be stronger if that distinction were made more explicit throughout.

Response 14

We thank the reviewer for the overall supportive assessment and all the above constructive comments and suggestions. We hope that these revisions have addressed the reviewer’s concerns.

## Reviewer #2

Summary

Song et al. propose a generalizable workflow to derive spatial colleague connectivity from national-scale Dutch job registry data covering over 8 million working individuals. The authors count undirected colleague pairs indexed by municipality triplets (two residential municipalities and one shared workplace municipality), then aggregate these to safety region and province levels. They demonstrate the epidemiological relevance of this connectivity by associating it with provincial SARS-CoV-2 Omicron onset timing using Bayesian hierarchical modelling, and they quantify how regional lockdowns and cross-regional travel bans would reduce total colleague links. A key finding is that a tenfold increase in within-province colleague connections is associated with Omicron onset approximately 12 days earlier (95% CI: 2–22 days), while between-province connections show a weaker and less certain association of 8 days earlier (95% CI: −4 to 21 days). The study also shows that the potential impact of regional lockdowns varies substantially by region, with locking down province Zuid-Holland removing up to 25.3% of all national colleague connections compared to only 2.6% for Zeeland.

Positive Feedback
• Utilizing large-scale payroll tax registrations to map a comprehensive national workplace network overcomes the representativeness biases inherent in commercial mobility/mobile phone datasets.
• The aggregated network datasets are openly accessible and providing reproducible R code on GitLab.
• The manuscript is well-written, logically organized, and effectively communicate complex spatial interactions.

Major Concerns

1. The degree cap of 100 colleagues per person is adopted from a prior CBS study (van der Laan et al., 2023) and exceeds the average of 13.4 daily contacts from POLYMOD by nearly eightfold. The authors justify this by appealing to “cumulative contact opportunities over longer time periods. Sensitivity results for caps of 50 and 200 are available in the online dataset but are not presented or discussed in the main text or supplementary figures. Since the cap directly determines the total number of connections (132 million at cap = 100) and shapes the regional connectivity patterns, these sensitivity results should be shown and discussed.

Response 15

We thank the reviewer for raising this crucial point. As also described in response 5 (Reviewer 1), we added the visualization and implications of the sensitivity analysis of the degree cap as a supplemental figure and added texts in the Result section where results of sensitivity analysis are presented:

*RESULTS: The spatial connectivity patterns ~~are stable~~* ***show broadly consistent province-level contrasts*** *across different choices of the degree cap*$D$*(i.e., the maximum number of colleague connections an individual is allowed to have****)****,* ***although the total number of inferred colleague links (ranged from 61 million to 239 million) and their spatial compositions changed with the choice of cap (supplementary Fig S8)*** *~~datasets presented online (3))~~.* ***As the cap increased from 50 to 200, the percentage of between-province connections rose by up to 5.56%, at the expense of within-municipality links.***

**Figure and captions added to the *Supplementary Information*:**

*"Fig S8: Sensitivity of spatial colleague connectivity patterns to the degree cap* $D$*.* *The figure shows the proportion of colleague links of the year 2022 that are within municipalities, within provinces, and between provinces under degree caps of 50, 100, and 200. As the degree cap increases, the proportion of within-municipality connections decreases by up to 5.24% (province ZL in the top panel), while the proportion of connections across provinces increases by up to 5.56% (province FL in the bottom panel). This pattern is expected, as using a higher degree cap allows for more connections in workplaces with many employees, which tend to connect individuals across wider geographical areas. Importantly, though the exact proportions per province across caps change, the contrast between provinces per cap remains similar."*

2. The regional intervention analyses the proportion of colleague connections removed under a lockdown or travel ban, but do not translate these reductions into expected epidemiological outcomes such as delayed Omicron onset. This is a missed opportunity, given that the regression model already provides an estimated relationship between log colleague connection counts and provincial onset timing. The authors could apply this relationship to post-intervention connection counts to produce a concrete epidemiological output. If a direct application of the regression is considered inappropriate (e.g., because the model is cross-sectional and the estimated association should not be interpreted causally as an intervention effect), the authors should at minimum provide a qualitative discussion of what the observed connectivity reductions would imply for transmission dynamics. As it stands, the intervention section describes the input to a policy decision how many links are severed without addressing the output that policymakers ultimately care about.

Response 16

We understand that the reviewer is aiming for stronger policy actionability based on the regional intervention analyses. However, we do not think it would be appropriate to extrapolate the estimated regression coefficient to predict delays in regional Omicron onset timing under counterfactual intervention scenarios for two reasons: (1) the regression model estimates an observed association between colleague connectivity and regional onset timing, but not the causal intervention effects; (2) changes in connectivity in one area (due to regional interventions) also change the connectivity patterns for other regions, as shown in Fig 5. Therefore, the epidemiological impact of a regional lockdown or travel ban cannot be represented as an isolated change in one province-level covariate. To appropriately predict the impact of interventions that affect colleague connectivity on infectious disease transmission, our data product would need to be used as an in input into a dynamic transmission model (and for which it is ultimately intended). We now state this more clearly in the *Discussion*:

*DISCUSSION:* *In conclusion, we establish a reusable data product of geographical connectedness in colleague networks****. This workplace connectivity provides a valuable spatial input for future transmission modelling and policy evaluation, particularly for representing how workplace links are distributed within and between regions.*** *and confirm its utility in designing and evaluating more regionally targeted and social-network tailored pandemic measures.* ***The Omicron analysis reassures that how such connectivity patterns are associated with regional differences in outbreak timing; further dynamic transmission modelling is needed to assess the impact of interventions that affect colleague connectivity.***

In addition, following the reviewer’s suggestion, we added a qualitative discussion in the *Discussion* section describing how reductions of connections may be interpreted in relation to transmission dynamics.

**Additions in the *Discussion* section:**

*"For instance, when infection levels are high in a specific area, regions that are strongly connected to it through workplace links may represent plausible candidates for enhanced surveillance or early-warning prioritisation. Among all preventive and control measures, mobility restrictions such as regional lockdowns or cross-regional travel bans may disrupt workplace links and thereby reduce potential workplace-related pathways through which infections could be seeded and spread onward to other regions."*

Minor Comments

1. The authors should acknowledge and cite the literature showing that commuting fluxes are a primary driver of epidemic onset timing, to better contextualise the choice of colleague connections as the measure of spatial connectivity instead of using other sources of mobility.

Response 17

We thank the reviewer for this suggestion. We have added the following sentence at the end of the first paragraph of the Introduction section to better contextualize the choice for colleague networks (additions in bold):

*INTRODUCTION: Within the wider population contact networks, colleague networks play a central role in shaping cross-regional population mixing due to their relatively higher volume compared to friend and family links and wider spatial reach compared to classmate and household connections (4).* ***Such work-related cross-regional commuting is an important channel for the spatial transmission of respiratory infectious diseases, including seasonal influenza and COVID-19* (5–7)*.***

2. Line 120: “most colleague pairs live within the sane province” should read “same province.”

Response 18

We thank the reviewer for this correction. We have corrected this typo in the manuscript.

3. The abstract and main text report slightly inconsistent confidence intervals for the association findings. The abstract states “12-day earlier (95% CI: 2 to 22 days)” for within-province connections, while the main text (Section 2.2, lines 165–168) reports “12 days earlier (95% CI: 2 to 21 days)”. Similarly, the between-province estimate is reported as “8-day earlier (95% CI: −4 to 21 days)” in the abstract and “8 days earlier (95% CI: −4 to 20 days)” in the main text. These discrepancies should be resolved; one consistent set of values should appear throughout the manuscript, abstract, and any supplementary materials.

Response 19

We thank the reviewer for pointing out this inconsistency. The discrepancy was due to a rounding error, which has now been corrected.

We have also revised the reporting of the slope parameters to improve interpretability. Specifically, instead of transforming the connection counts to logarithmic scale with a natural base and standardize (from equation 9 to 11), we now use a logarithmic transformation with base 2 and report the estimated slope parameters on the non-standardized base-2 logarithmic scale. The estimate therefore represents the change in Omicron onset timing associated with a twofold increase in within- or between-province colleague connections, or inferred colleague links with Noord-Holland. This avoids reporting coefficients on a standardized logarithmic scale and makes the magnitude directly interpretable. This has not changed the substantial contents of the results or the conclusions.

We revised the Method section where the logarithmic transformation and standardization was described, the Abstract and Result sections where the twofold changes of inferred colleague links are mentioned (additions in bold, removed texts with a strikethrough).

*METHODS*:

*For each province* $i,$*we summarized the connectivity derived in Section 2.2 to quantify the level of internal and external links. We decomposed links per residential province into two directional categories: (1) within-province links where both individuals live and work in province* $i$*, denoted as* $d_{i}^{\text{wt}}$*; (2) between-province links where at least one individual resides or works in another province, denoted as* $d_{i}^{\text{bt}}$*. Because multiplicative changes are more meaningful compared to additive changes in colleague connections, we worked with a logarithmic scale of the two types of connections and then standardized across provinces for better Hamilton Monte Carlo sampling efficiency,*

$$\tilde{d_{i}^{\text{wt}}}=\frac{\text{log}_{\boldsymbol{2}} \left( d_{i}^{\text{wt}} \right)-\text{mean}\left( \text{log}_{\boldsymbol{2}} \left( d_{i}^{wt} \right) \right)}{\text{SD}\left( \text{log}_{\boldsymbol{2}} \left( d_{i}^{wt} \right) \right)}$$

$$\tilde{d_{i}^{\text{bt}}}=\frac{\text{log}_{2} \left( d_{i}^{bt} \right)-\text{mean}\left( \text{log}_{\boldsymbol{2}} \left( d_{i}^{bt} \right) \right)}{\text{SD}\left( \text{log}_{\boldsymbol{2}} \left( d_{i}^{\text{bt}} \right) \right)}$$

*We then fit the model for each draw of the estimated Omicron onset time for province* $i$

$$\begin{aligned} \hat{T}_{i}^{\left( \mathcal{l} \right)}=\alpha_{1}+\beta^{\text{wt}}\tilde{\boldsymbol{\beta}^{\text{wt}}}\cdot\tilde{d_{i}^{\text{wt}}}+\beta^{BT}\tilde{\boldsymbol{\beta}^{\text{bt}}}\cdot\tilde{d_{i}^{\text{bt}}}, \mathcal{l=}1,2,\ldots, 1000\#\left( 10 \right) \end{aligned}$$

***where coefficients*** $\tilde{\boldsymbol{\beta}^{\text{wt}}}$ ***and*** $\tilde{\boldsymbol{\beta}^{\text{bt}}}$ ***represent the association with Omicron onset timing per one standard deviation increase in*** $\text{log}_{\boldsymbol{2}} \left( \boldsymbol{d}_{\boldsymbol{i}}^{\boldsymbol{bt}} \right)$ ***and*** $\text{log}_{\boldsymbol{2}} \left( \boldsymbol{d}_{\boldsymbol{i}}^{\boldsymbol{bt}} \right)$***. To report the slopes on the unstandardized logarithmic scale, we transformed the coefficients as***

$$\boldsymbol{\beta}^{\text{wt}}\boldsymbol{=}\frac{\tilde{\boldsymbol{\beta}^{\text{wt}}}}{\text{SD }\left( \boldsymbol{log}_{\boldsymbol{2}} \left( \boldsymbol{d}_{\boldsymbol{i}}^{\text{wt}} \right) \right)}$$

$$\boldsymbol{\beta}^{\boldsymbol{bt}}\boldsymbol{=}\frac{\tilde{\boldsymbol{\beta}^{\text{bt}}}}{\text{SD }\left( \text{log}_{\boldsymbol{2}} \left( \boldsymbol{d}_{\boldsymbol{i}}^{\text{bt}} \right) \right)}$$

***Therefore,*** $\boldsymbol{\beta}^{\text{wt}}$ ***and*** $\boldsymbol{\beta}^{\text{bt}}$***represent the change in Omicron onset timing associated with a twofold increase in within-province and between-province colleague connections, respectively. Since onset timing was measured in weeks, we multiplied these transformed slopes by 7 to report the associations in days.***

*As Omicron was first seeded in the Netherlands through international flights from South Africa to the Netherlands, the onset of Omicron in Noord-Holland, where Amsterdam Airport Schiphol locates, was driven by international transmissions instead of within-country mixing. This makes Noord-Holland a central complication for interpreting the plausible role of domestic colleague connectivity in shaping regional outbreak dynamics. We additionally tested whether stronger links to Noord-Holland, as a proxy for proximity to the primary importation hub, were associated with earlier local onset elsewhere. We computed for each residential province, number of colleague connections with individuals living in Noord-Holland, denoted as* $d_{i}^{\text{NH}}$*. Similarly, we first standardized the logarithmic of* $d_{i}^{\text{NH}}$ *for sampling efficiency:*

$$\tilde{d_{i}^{\text{NH}}}=\frac{\text{log}_{\boldsymbol{2}} \left( d_{i}^{\text{NH}} \right)-\text{mean}\left( \text{log}_{\boldsymbol{2}} \left( d_{i}^{\text{NH}} \right) \right)}{\text{SD }\left( \text{log}_{\boldsymbol{2}} \left( d_{i}^{\text{NH}} \right) \right)}$$

*We fit the second model as:*

$\begin{aligned} \hat{T}_{i}^{\left( \mathcal{l} \right)}=\alpha_{2}+\tilde{\beta^{\text{NH}}}\cdot\tilde{d_{i}^{\text{NH}}},\mathcal{l=}1,2,\ldots, 1000\#\#\left( 11 \right) \end{aligned}$

***Again, we transform the coefficient to the unstandardized logarithmic scale as***

$$\boldsymbol{\beta}^{\text{NH}}\boldsymbol{=}\frac{\tilde{\boldsymbol{\beta}^{\text{NH}}}}{\boldsymbol{SD}\left( \boldsymbol{log}_{\boldsymbol{2}} \left( \boldsymbol{d}_{\boldsymbol{i}}^{\text{NH}} \right) \right)}$$

*RESULT* and *ABSTRACT regarding Fig 4*:

*~~A negative slope of -0.62 on the standardized natural logarithmic scale with base 2 (Fig 4) indicates that a tenfold increase in within-province colleague connections is associated with an Omicron onset that is 12 days earlier (95% CI: 2 to 21 days earlier). Similarly, the between-province connectivity slope of -0.36 suggests that a tenfold increase in inter-province colleague connections is associated with an Omicron onset that is 8 days earlier (95% CI: -4 to 20 days earlier).~~* ***As shown in Fig 4, a two-fold increase in within-province colleague connections was associated with an Omicron onset that was 3.7 days earlier (95% CI: 0.6 to 6.6 days), while a two-fold increase in between-province colleague connections was associated with an onset that was 2.5 days earlier (95% CI: -1.0 to 6.2 days).***

Updated Fig 4. Associations between regional colleague connectivity and Omicron onset timing.

*Supplementary Information regarding Fig S5:*

*Association between connectivity with Noord-Holland and Omicron onset time. ~~A negative association indicates provinces with more colleague connections with Noord-Holland are associated with earlier Omicron onset. A negative slope of -0.53 on the standardized natural logarithmic scale indicates that a tenfold increase in within-province colleague connections is associated with an Omicron onset that is, on average, 7 days earlier.~~* ***A two-fold increase in colleague connections with residents of Noord-Holland was associated with an onset that was 2.0 days earlier (95% CI: 0.0 to 4.1 days).*** *Full names of provinces and the corresponding abbreviations are shown in supplementary Table S1.2.*

Updated Fig S5. Association between connectivity with Noord-Holland and Omicron onset timing.

4. The Data and Code Availability statement (line 675) contains a typo: “resgitry” should read “registry.”

Response 20

We have corrected this typo in the manuscript.

## References

1.	van der Laan J, de Jonge E, Das M, Te Riele S, Emery T. A Whole Population Network and Its Application for the Social Sciences. Eur Sociol Rev. 2023 Feb;39(1):145–60. doi:10.1093/esr/jcac026

2.	Dunbar RIM. Neocortex size as a constraint on group size in primates. Journal of Human Evolution. 1992 Jun;22(6):469–93. doi:10.1016/0047-2484(92)90081-J

3.	Song P, de Vlas SJ, Emery T, Coffeng LE. Municipality-level geographical colleague connectivity in the Netherlands (2019-2022) [Internet]. DataverseNL; 2026 [cited 2026 Jan 18]. Available from: https://dataverse.nl/dataset.xhtml?persistentId=doi:10.34894/XF7IAD doi:10.34894/XF7IAD

4.	Feehan DM, Mahmud AS. Quantifying population contact patterns in the United States during the COVID-19 pandemic. Nat Commun. 2021 Feb;12(1):893. doi:10.1038/s41467-021-20990-2

5.	Charaudeau S, Pakdaman K, Boëlle PY. Commuter Mobility and the Spread of Infectious Diseases: Application to Influenza in France. Vespignani A, editor. PLoS ONE. 2014 Jan;9(1):e83002. doi:10.1371/journal.pone.0083002

6.	Mitze T, Kosfeld R. The propagation effect of commuting to work in the spatial transmission of COVID-19. J Geogr Syst. 2022 Jan;24(1):5–31. doi:10.1007/s10109-021-00349-3

7.	Charu V, Zeger S, Gog J, Bjørnstad ON, Kissler S, Simonsen L, et al. Human mobility and the spatial transmission of influenza in the United States. PLoS Comput Biol. 2017 Feb;13(2):e1005382. doi:10.1371/journal.pcbi.1005382 PubMed PMID: 28187123; PubMed Central PMCID: PMC5349690.
