# Supplementary material for: Mapping spatial colleague connectivity patterns from individual-level registry data to inform regional pandemic interventions
Source: PLoS Comput Biol. 2026 Aug 31;22(8):e1014721. doi: 10.1371/journal.pcbi.1014721 (PMC13568526; doi:10.1371/journal.pcbi.1014721)
Supplement: Peer review history

# Peer review: Decision Letter 0

**DOI:** 10.1371/journal.pcbi.1014721.r001

**Authors:** Jennifer Flegg, Nicholas Geard

9 Jun 2026

Mapping spatial colleague connectivity patterns from individual-level registry data to inform regional pandemic interventions

PLOS Computational Biology

Dear Dr. Song,

Thank you for submitting your manuscript to PLOS Computational Biology. After careful consideration, we feel that it has merit but does not fully meet PLOS Computational Biology's publication criteria as it currently stands. Therefore, we invite you to submit a revised version of the manuscript that addresses the points raised during the review process.

Please submit your revised manuscript by Aug 09 2026 11:59PM. If you will need more time than this to complete your revisions, please reply to this message or contact the journal office at ploscompbiol@plos.org. When you're ready to submit your revision, log on to https://www.editorialmanager.com/pcompbiol/ and select the 'Submissions Needing Revision' folder to locate your manuscript file.

Please include the following items when submitting your revised manuscript:

* A letter that responds to each point raised by the editor and reviewer(s). You should upload this letter as a separate file labeled 'Response to Reviewers'. This file does not need to include responses to formatting updates and technical items listed in the 'Journal Requirements' section below.

* A marked-up copy of your manuscript that highlights changes made to the original version. You should upload this as a separate file labeled 'Revised Manuscript with Track Changes'.

* An unmarked version of your revised paper without tracked changes. You should upload this as a separate file labeled 'Manuscript'.

Kind regards,

Nicholas Geard

Academic Editor

PLOS Computational Biology

Jennifer Flegg

Section Editor

PLOS Computational Biology

**Additional Editor Comments :**

Thank you for your submission. The reviewers agree that your manuscript makes a novel and valuable methodological contribution, but overstates some of the epidemiological interpretations drawn from the Omicron case study. The reviewers have provided detailed suggestions about how the language of the manuscript could be modified to provide a more nuanced assessment of the proposed approach to modelling workplace contacts without lessening the impact of your study. I encourage you to consider and address them carefully in your revision.

**Journal Requirements:**

1) Please ensure that the CRediT author contributions listed for every co-author are completed accurately and in full.

At this stage, the following Authors/Authors require contributions: PingPing Song, Sake J. de Vlas, Tom Emery, and Luc E. Coffeng. Please ensure that the full contributions of each author are acknowledged in the "Add/Edit/Remove Authors" section of our submission form.

The list of CRediT author contributions may be found here: https://journals.plos.org/ploscompbiol/s/authorship#loc-author-contributions

2) We ask that a manuscript source file is provided at Revision. Please upload your manuscript file as a .doc, .docx, .rtf or .tex. If you are providing a .tex file, please upload it under the item type u2018LaTeX Source Fileu2019 and leave your .pdf version as the item type u2018Manuscriptu2019.

3) Please upload all main figures as separate Figure files in .tif or .eps format. For more information about how to convert and format your figure files please see our guidelines:

https://journals.plos.org/ploscompbiol/s/figures

4) Please upload a copy of Figure 1 to 8 which you refer to in your text  on the manuscript file. Or, if the figure is no longer to be included as part of the submission please remove all reference to it within the text.

5) Thank you for stating "Genome sequences and associated metadata are published in GISAID's EpiCoV database with the GISAID identifier being EPI_SET_251126rq, and openly accessible(38)"

Please note that your Data Availability Statement is currently missing a direct link to access the database]. If your manuscript is accepted for publication, you will be asked to provide these details on a very short timeline. We therefore suggest that you provide this information now, though we will not hold up the peer review process if you are unable.

6) Thank you fo rstating "The aggregated colleague connectivity (i.e. colleague pair counts per municipality triplet that this study generates) derived from country-wide resgitry data are openly accessible, together with the code that could reproduce the data within CBS Microdata environment(23). However, the individual-level registry data are not publicly available and can only be accessible upon request to CBS Microdata."

Please provide contact information  to which data requests may be sent.

7) Please amend your detailed Financial Disclosure statement. This is published with the article, therefore should be completed in full sentences and contain the exact wording you wish to be published.

i). State what role the funders took in the study. If the funders had no role in your study, please state: “The funders had no role in study design, data collection and analysis, decision to publish, or preparation of the manuscript.”

ii). If any authors received a salary from any of your funders, please state which authors and which funders.

8) We have amended your Competing Interest statement to comply with journal style. We kindly ask that you double check the statement and let us know if anything is incorrect.

**Reviewers' comments:**

Reviewer's Responses to Questions

**Comments to the Authors:**

**Please note here if the review is uploaded as an attachment.**

Reviewer #1: See attached document with my thoughts.

Reviewer #2: Summary

Song et al. propose a generalizable workflow to derive spatial colleague connectivity from national-scale Dutch job registry data covering over 8 million working individuals. The authors count undirected colleague pairs indexed by municipality triplets (two residential municipalities and one shared workplace municipality), then aggregate these to safety region and province levels. They demonstrate the epidemiological relevance of this connectivity by associating it with provincial SARS-CoV-2 Omicron onset timing using Bayesian hierarchical modelling, and they quantify how regional lockdowns and cross-regional travel bans would reduce total colleague links. A key finding is that a tenfold increase in within-province colleague connections is associated with Omicron onset approximately 12 days earlier (95% CI: 2–22 days), while between-province connections show a weaker and less certain association of 8 days earlier (95% CI: −4 to 21 days). The study also shows that the potential impact of regional lockdowns varies substantially by region, with locking down province Zuid-Holland removing up to 25.3% of all national colleague connections compared to only 2.6% for Zeeland.

Positive Feedback

• Utilizing large-scale payroll tax registrations to map a comprehensive national workplace network overcomes the representativeness biases inherent in commercial mobility/mobile phone datasets.

• The aggregated network datasets are openly accessible and providing reproducible R code on GitLab.

• The manuscript is well-written, logically organized, and effectively communicate complex spatial interactions.

Major Concerns

• The degree cap of 100 colleagues per person is adopted from a prior CBS study (van der Laan et al., 2023) and exceeds the average of 13.4 daily contacts from POLYMOD by nearly eightfold. The authors justify this by appealing to “cumulative contact opportunities over longer time periods. Sensitivity results for caps of 50 and 200 are available in the online dataset but are not presented or discussed in the main text or supplementary figures. Since the cap directly determines the total number of connections (132 million at cap = 100) and shapes the regional connectivity patterns, these sensitivity results should be shown and discussed.

● The regional intervention analyses the proportion of colleague connections removed under a lockdown or travel ban, but do not translate these reductions into expected epidemiological outcomes such as delayed Omicron onset. This is a missed opportunity, given that the regression model already provides an estimated relationship between log colleague connection counts and provincial onset timing. The authors could apply this relationship to post-intervention connection counts to produce a concrete epidemiological output. If a direct application of the regression is considered inappropriate (e.g., because the model is cross-sectional and the estimated association should not be interpreted causally as an intervention effect), the authors should at minimum provide a qualitative discussion of what the observed connectivity reductions would imply for transmission dynamics. As it stands, the intervention section describes the input to a policy decision how many links are severed without addressing the output that policymakers ultimately care about.

Minor Comments

• The authors should acknowledge and cite the literature showing that commuting fluxes are a primary driver of epidemic onset timing, to better contextualise the choice of colleague connections as the measure of spatial connectivity instead of using other sources of mobility.

• Line 120: “most colleague pairs live within the sane province” should read “same province.”

• The abstract and main text report slightly inconsistent confidence intervals for the association findings. The abstract states “12-day earlier (95% CI: 2 to 22 days)” for within-province connections, while the main text (Section 2.2, lines 165–168) reports “12 days earlier (95% CI: 2 to 21 days)”. Similarly, the between-province estimate is reported as “8-day earlier (95% CI: −4 to 21 days)” in the abstract and “8 days earlier (95% CI: −4 to 20 days)” in the main text. These discrepancies should be resolved; one consistent set of values should appear throughout the manuscript, abstract, and any supplementary materials.

• The Data and Code Availability statement (line 675) contains a typo: “resgitry” should read “registry.”

**********

**Have the authors made all data and (if applicable) computational code underlying the findings in their manuscript fully available?**

The PLOS Data policy requires authors to make all data and code underlying the findings described in their manuscript fully available without restriction, with rare exception (please refer to the Data Availability Statement in the manuscript PDF file). The data and code should be provided as part of the manuscript or its supporting information, or deposited to a public repository. For example, in addition to summary statistics, the data points behind means, medians and variance measures should be available. If there are restrictions on publicly sharing data or code —e.g. participant privacy or use of data from a third party—those must be specified.

Reviewer #1: **No:** As stated in their paper, some individual-level data is not available. However, they provide a repository with enough data and code to reproduce the example within their environment.

Reviewer #2: Yes

**********

PLOS authors have the option to publish the peer review history of their article (what does this mean?). If published, this will include your full peer review and any attached files.

If you choose “no”, your identity will remain anonymous but your review may still be made public.

**Do you want your identity to be public for this peer review?**  For information about this choice, including consent withdrawal, please see our Privacy Policy.

Reviewer #1: No

Reviewer #2: No

[NOTE: If reviewer comments were submitted as an attachment file, they will be attached to this email and accessible via the submission site. Please log into your account, locate the manuscript record, and check for the action link "View Attachments". If this link does not appear, there are no attachment files.]

**Figure resubmission:**

**Reproducibility:**

?>

Attachment Submitted filename: Review comments.docx

# Author comment: Author response to Decision Letter 1

**DOI:** 10.1371/journal.pcbi.1014721.r002

4 Aug 2026

Attachment Submitted filename: Reply to reviewers.docx

# Peer review: Decision Letter 1

**DOI:** 10.1371/journal.pcbi.1014721.r003

**Authors:** Jennifer Flegg, Nicholas Geard

17 Aug 2026

Dear Miss Song,

We are pleased to inform you that your manuscript 'Mapping spatial colleague connectivity patterns from individual-level registry data to inform regional pandemic interventions' has been provisionally accepted for publication in PLOS Computational Biology.

Before your manuscript can be formally accepted you will need to complete some formatting changes, which you will receive in a follow up email. A member of our team will be in touch with a set of requests.

Please note that your manuscript will not be scheduled for publication until you have made the required changes, so a swift response is appreciated.

IMPORTANT: The editorial review process is now complete. PLOS will only permit corrections to spelling, formatting or significant scientific errors from this point onwards. Requests for major changes, or any which affect the scientific understanding of your work, will cause delays to the publication date of your manuscript.

Should you, your institution's press office or the journal office choose to press release your paper, you will automatically be opted out of early publication. We ask that you notify us now if you or your institution is planning to press release the article. All press must be co-ordinated with PLOS.

Thank you again for supporting Open Access publishing; we are looking forward to publishing your work in PLOS Computational Biology.

Best regards,

Nicholas Geard

Academic Editor

PLOS Computational Biology

Jennifer Flegg

Section Editor

PLOS Computational Biology

***********************************************************

Reviewer's Responses to Questions

**Comments to the Authors:**

**Please note here if the review is uploaded as an attachment.**

Reviewer #2: Authors answered carefully all my concerns. I recommend this paper for publication.

**********

**Have the authors made all data and (if applicable) computational code underlying the findings in their manuscript fully available?**

The PLOS Data policy requires authors to make all data and code underlying the findings described in their manuscript fully available without restriction, with rare exception (please refer to the Data Availability Statement in the manuscript PDF file). The data and code should be provided as part of the manuscript or its supporting information, or deposited to a public repository. For example, in addition to summary statistics, the data points behind means, medians and variance measures should be available. If there are restrictions on publicly sharing data or code —e.g. participant privacy or use of data from a third party—those must be specified.

Reviewer #2: Yes

**********

PLOS authors have the option to publish the peer review history of their article (what does this mean?). If published, this will include your full peer review and any attached files.

If you choose “no”, your identity will remain anonymous but your review may still be made public.

**Do you want your identity to be public for this peer review?**  For information about this choice, including consent withdrawal, please see our Privacy Policy.

Reviewer #2: No

# Editor report: Acceptance letter

**DOI:** 10.1371/journal.pcbi.1014721.r004

**Authors:** Jennifer Flegg, Nicholas Geard

PCOMPBIOL-D-26-00784R1

Mapping spatial colleague connectivity patterns from individual-level registry data to inform regional pandemic interventions

Dear Dr Song,

I am pleased to inform you that your manuscript has been formally accepted for publication in PLOS Computational Biology. Your manuscript is now with our production department and you will be notified of the publication date in due course.

The corresponding author will soon be receiving a typeset proof for review, to ensure errors have not been introduced during production. Please review the PDF proof of your manuscript carefully, as this is the last chance to correct any errors. Please note that major changes, or those which affect the scientific understanding of the work, will likely cause delays to the publication date of your manuscript.

Soon after your final files are uploaded, unless you have opted out, the early version of your manuscript will be published online. The date of the early version will be your article's publication date. The final article will be published to the same URL, and all versions of the paper will be accessible to readers.

For Research, Software, and Methods articles, you will receive an invoice from PLOS for your publication fee after your manuscript has reached the completed accept phase. If you receive an email requesting payment before acceptance or for any other service, this may be a phishing scheme. Learn how to identify phishing emails and protect your accounts at https://explore.plos.org/phishing.

Thank you again for supporting PLOS Computational Biology and open-access publishing. We are looking forward to publishing your work!

With kind regards,

Sharmila Kamatchi

PLOS Computational Biology | Carlyle House, Carlyle Road, Cambridge CB4 3DN | United Kingdom ploscompbiol@plos.org | Phone +44 (0) 1223-442824 | ploscompbiol.org | @PLOSCompBiol
